# SGLT2i and GLP1-RA exert additive cardiorenal protection with a RAS blocker in uninephrectomized db/db mice

**DOI:** 10.3389/fphar.2024.1415879

**Published:** 2024-10-07

**Authors:** Nerea Martos-Guillami, Ander Vergara, Carmen Llorens-Cebrià, Aku Enam Motto, Irene Martínez-Díaz, Francisco Gonçalves, Maria Magdalena Garcias-Ramis, Estibaliz Allo-Urzainqui, Alonso Narváez, Sheila Bermejo, Vicent Muñoz, Juan León-Román, Roser Ferrer-Costa, Conxita Jacobs-Cachá, Jordi Vilardell-Vilà, María José Soler

**Affiliations:** ^1^ Nephrology and Transplantation Research Group, Vall d’Hebron Institut de Recerca (VHIR), Nephrology Department, Vall d’Hebron Hospital Universitari, Vall d’Hebron Barcelona Hospital Campus, Barcelona, Spain; ^2^ Redes de Investigación Cooperativa Orientadas a Resultados en Salud (RICORS), Instituto de Salud Carlos III (RD21/0005/0016), Madrid, Spain; ^3^ Laboratory of Physiology/ Pharmacology, Unit of Pathophysiology, Bioactive Substances and Safety, Faculty of Sciences, University of Lomé, Lomé, Togo; ^4^ Clinical Biochemistry Department, Vall d’Hebron Hospital Universitari, Vall d’Hebron Barcelona Hospital Campus. Barcelona, Barcelona, Spain; ^5^ Urology Department, Vall d’Hebron Hospital Universitari, Vall d’Hebron Barcelona Hospital Campus, Barcelona, Spain

**Keywords:** diabetes, chronic kidney disease (CKD), diabetic kidney disease (DKD), sodium-glucose cotransporter 2 inhibitors (SGLT2i), glucagon-like-1 receptor agonists (GLP-1RA), renin-angiotensin system (RAS)

## Abstract

**Introduction:**

Diabetic Kidney Disease (DKD) is the main cause of end-stage renal disease in the developed world. The current treatment of the DKD with renin-angiotensin system (RAS) blockade does not totally halt the progression to end stage kidney disease. Currently, several drugs have shown to delay DKD progression such as sodium-glucose co-transporter-2 inhibitors (SGLT2i) and glucagon-like-1 receptor agonists (GLP-1RA). We hypothesized that by combining several drugs that prevent DKD progression on top of RAS blockade a synergistic effect would be achieved in terms of cardiorenal protection. In the present study, we analysed if the combination of a RAS blocker (ramipril) with a SGLT2i (empagliflozin) and/or GLP-1RA (semaglutide) in a type 2 diabetic mouse model could have add-on effects in kidney and heart protection.

**Methods:**

Male and female uninephrectomized type 2 diabetic db/db mice were treated with empagliflozin and/or semaglutide on top of ramipril during 8 weeks. During the study body weight, water and food intake were weekly monitored, glycaemia biweekly and albuminuria and glomerular filtration rate (GFR) before and after the treatment. At the end of the experiment, kidney and heart were isolated for histological and gene expression studies as well as for intrarenal RAS state assessment.

**Results:**

Semaglutide combined with ramipril and/or empagliflozin significantly decreased albuminuria but only when combined with both compounds, semaglutide further decreased blood glucose, glomerular hyperfiltration in male mice and glomerular mesangial matrix expansion. In kidney, only the triple treatment with empagliflozin, semaglutide and ramipril reduced the expression of the proinflammatory and profibrotic genes ccl2 and TGFß1. In addition, the combination of empagliflozin and semaglutide on top of RAS blockade was superior in decreasing cardiomyocyte hypertrophy and heart fibrosis in db/db mice.

**Discussion:**

Our results suggest that the combination of SGLT2i with GLP-1RA is superior in cardiorenal protection in DKD than the drugs administered alone on top of RAS blockade.

## 1 Introduction

Diabetic kidney disease (DKD) is the main cause of end-stage renal disease in the developed world; its mortality is higher than that registered for breast or colorectal cancer patients (Ruggenenti et al., 2008). DKD is defined as a chronic kidney disease (CKD) attributable to diabetes and characterized by an estimated glomerular filtration rate (eGFR) < 60 mL/min/1.73 m^2^ or a urinary albumin-to-creatinine ratio (UACR) ≥ 30 mg albumin/g creatinine. Up to 35% of DKD patients have decreased renal function with albuminuria A1 (UACR <30 mg/g) or albuminuria A2, but DKD patients with UACR >300 (A3 albuminuria) have the highest risk of disease progression and premature death (Fernandez-Fernandez et al., 2019). The classical therapies for DKD are focused on controlling hyperglycemia and blood pressure mainly by administering renin-angiotensin system (RAS) blockers together with supplementary treatments like statins, a low-salt diet, and smoking cessation (Kidney Disease: Improving Global Outcomes KDIGO Diabetes Work Group, 2022). Despite optimal clinical management of the disease, the current conservative treatment of the DKD does not completely halt the progression to end-stage kidney disease. Therefore, novel therapeutic strategies are most important (Ruggenenti et al., 2008; Kidney Disease: Improving Global Outcomes KDIGO Diabetes Work Group, 2022).

In 2014, several drugs emerged with the potential to delay DKD. The endothelin receptors subtype A antagonists (ERAs) reduced the progression of DKD (de Zeeuw et al., 2014; Heerspink et al., 2019). However, ERAs had unwanted side effects, such as favoring heart failure by increasing anemia and fluid retention (Heerspink et al., 2019; Wenzel et al., 2009), that have limited their clinical use. Recently, the Fidelio and Figaro-DKD randomized clinical trials (RCT) showed that finerenone, a selective nonsteroidal mineralocorticoid receptor antagonist, reduced the risk of CKD progression and cardiovascular events compared to placebo in patients with diabetes and CKD (Bakris et al., 2020; Pitt et al., 2021). Further, sodium-glucose cotransporter-2 inhibitors (SGLT2i) that are widely used to control blood glucose levels have demonstrated renoprotective effects (García-Carro et al., 2019; Martínez-Díaz et al., 2023). SGLT2i acts on SGLT2, a Na+/glucose cotransporter present in the proximal tubular cells of the kidney. Under normal conditions, SGLT2 maintains glucose homeostasis and the intraglomerular tone by reabsorbing sodium and glucose present in the lumen of the tubule and allowing their subsequent return into the bloodstream (Szablewski, 2017; Navale and Paranjape, 2016). In patients with diabetes, hyperglycemia, and hypertension, the treatment with SGLT2i blocks this reabsorption mechanism, increasing natriuresis and ultimately reducing the intraglomerular pressure. Interestingly, the SGLT2i appears to protect patients with type 2 diabetes and CKD in combination with a RAS blocker. Empagliflozin, an SGLT2i, was evaluated in the EMPA-REG OUTCOME trial and showed a reductive risk of major adverse cardiovascular events in patients with type 2 diabetes (T2D) (Wanner et al., 2016). Surprisingly, an initial eGFR dip of over 10% after initiating SGLT2i did not have any impact on the subsequent cardiovascular death, hospitalization for heart failure, and incident or worsening kidney disease and demonstrated beneficial effects in people with heart failure and reduced ejection fraction (Kraus et al., 2021; Adamson et al., 2022). Further, several preclinical and clinical studies have demonstrated that dual therapy with RAS blockers and SGLT2i slows the progression of CKD in people with type 2 diabetes (Wanner et al., 2016; Górriz et al., 2020a; Mosenzon et al., 2019; Perkovic et al., 2019). In contrast to ERAs, SGLT2i has diuretic effects (Griffin et al., 2020; Vergara et al., 2019), increases hemoglobin levels (Lorenzo et al., 2023), and reduces the risk of heart failure (Anker et al., 2021; McMurray et al., 2019). In this sense, different studies have shown that the combination of SGLT2i with ERAs could present synergistic protective effects in terms of albuminuria decrease and less fluid retention (Heerspink et al., 2023; Heerspink et al., 2021; Vergara et al., 2022).

The administration of other hypoglycemic drugs, like glucagon-like-1 receptor agonists (GLP-1RA), decreases persistent macroalbuminuria in patients with DKD (Mann et al., 2017; Marso et al., 2016). In people with T2D, GLP-1RA reduces the incidence of sustained eGFR (Gerstein et al., 2019). Semaglutide, a GLP-1RA, interacts with the GLP-1 receptor, which results in multiple metabolic effects like insulin secretion, reductions of gastric emptying, and effects on the RAS pathway (Górriz et al., 2020b; Zander et al., 2002). The SUSTAIN-6 clinical trial has shown that semaglutide significantly improves glycemic control in patients with T2D (Sorli et al., 2017). The efficacy and safety of the combination of semaglutide and an SGLT2i in T2D patients were investigated in the SUSTAIN 9 RCT. The study demonstrated a reduction in both glycosylated hemoglobin (HbA1c) levels and body weight (Zinman et al., 2019). Recently, the FLOW study has demonstrated that semaglutide reduces major kidney disease events in people with T2D and CKD (Perkovic et al., 2024). These studies show that we already have new strategies to delay DKD progression and suggest that additional renoprotection can be achieved by combining different drug classes.

In the present study, we analyzed the synergistic cardiorenal protective effects of combining a RAS blocker (ramipril) with an SGLT2i (empagliflozin) and a GLP-1RA (semaglutide) in a mouse model of type 2 diabetes (db/db). Our main objective was to study whether SGLT2i combined with GLP-1RA showed superior cardiorenal benefits compared to SGLT2i or GLP-1RA in monotherapy combined with a RAS blocker.

## 2 Materials and methods

### 2.1 Animal model and experimental design

Eight-week-old male and female leptin-receptor diabetic mice (db/db) and non-diabetic heterozygous littermates (db/m) were purchased from Charles River (BKS.Cg-Dock7^m^ +/+ Lepr^db^J mice, strain code: 607). Mice were housed in an environmentally controlled room at constant temperature and humidity (22°C ± 2°C) under a 12 h light/dark cycle and had free access to standard chow and tap water. All mice were uninephrectomized at 6–7 weeks old to promote hyperfiltration (Ninichuk et al., 2007; Maekawa et al., 2022). At 12 weeks old, db/db mice were randomly assigned to four treatment arms for 8 weeks: 1) db/db mice treated with vehicle (n = 10 male and 4 female), 2) db/db mice treated with empagliflozin and ramipril (EMP + RAM, n = 9 male and 5 female), 3) db/db mice treated with semaglutide and ramipril (SEM + RAM, n = 7 male and 6 female), 4) db/db mice with a triple therapy treatment of empaglifozin, semaglutide, and ramipril (EMP + SEM + RAM, n = 9 male and 7 female). Non-diabetic mice (db/m) treated with vehicle were used as non-diabetic controls (n = 8 male and 5 female). The dose of empagliflozin (10 mg/kg/day) and ramipril (8 mg/kg/day) were the same as that previously published by our team (Vergara et al., 2022; Vergara et al., 2023). The semaglutide dose (10 nmol/kg diluted in PBS1x) was selected according to results obtained by other authors using similar mouse models (Nestor et al., 2022; Jiang et al., 2023). Empagliflozin diluted in 0.5% hydroxyethyl-cellulose was administrated by oral gavage 5 days per week, semaglutide was given twice per week subcutaneously, and ramipril was diluted in the drinking water. The mice in group B were subcutaneously injected with PBS1x twice per week, and the mice in group C were administered 0.5% hydroxyethyl-cellulose 5 days per week by oral gavage. Further, vehicle-treated mice received tap water, were subcutaneously injected with PBS1x twice a week, and 0.5% hydroxyethyl-cellulose was given 5 days per week by oral gavage. The body weight of the animals was measured weekly, blood glucose was measured biweekly, and UACR, glomerular filtration rate (GFR), and blood pressure were measured before and 8 weeks post-treatment. At the end of the study, animals were anesthetized using pentobarbital (100 mg/g intraperitoneal bolus). Once anesthetized, blood was obtained by cardiac puncture and collected in a micro-sample tube with clotting activator gel (41.1378.005, Sarstedt). Serum was obtained by centrifugation of the blood (2500 g, 10 min, 20°C) and stored at −80°C until use. Kidneys and hearts were removed for molecular (snap frozen tissue) and histology analysis (10% formalin-fixed and paraffin-embedded tissue).

The animal study protocol was approved by the Ethics Committee of our institution (Project number 59.21) and followed the European Council Directives for the care of animals used for research (2010/63/EU).

### 2.2 Weight, blood glucose, and blood pressure monitoring

Body weight was measured weekly from 9 weeks of age until the end of the experiment. Blood glucose was measured after 6 h of fasting, immediately before starting the treatment, and each 2 weeks after treatment initiation (at 12 weeks, 15 weeks, 17 weeks, and 19 weeks of age) using a hand-held glucometer (Accu-Chek Performa, Roche). Some blood samples were higher than the upper detection limit of the glucometer; in these cases, we considered the maximum possible value (600 mg/dL). Blood pressure (BP) was measured non-invasively in conscious mice using the tail-cuff method (LE 5001, Harvard Apparatus) before starting and after 8 weeks of treatment.

### 2.3 Computed tomography studies

Total body volume and body fat volume were measured before and after the treatment by microcomputed tomography using the Quantum FX imaging system (Perkin Elmer) under isoflurane anesthesia. Data obtained were analyzed by the Preclinical Imaging Platform staff at the Vall d’Hebron Research Institute. All the images obtained have a voxel size of 3.2418 µm^3^. To calculate the whole-body volume, a segmentation of complete body voxels from air voxels (minimum threshold −400 Hounsfield Units (HU)) was performed. Fat tissue was measured with a second segmentation of fat voxels (range −200–100 HU) (Wanner et al., 2016; Neal et al., 2017). Every segmentation was checked for proper fitting with the fat tissue. A local segmentation between intraabdominal and subcutaneous fat tissues was obtained by applying a “seeds and frontiers” method.

### 2.4 Transcutaneous GFR measurement

GFR was measured in mice before and after treatment by transcutaneous measurement of fluorescein, isothiocyanate (FITC)-sinistrin. Briefly, mouse skin was shaved, and a fluorescent signal measuring device (Transdermal GFR monitor, MediBeacon) was attached with an adhesive patch. The background signal of the skin was recorded for 5 min, and afterward, an intravenous bolus of a fluorescent agent (FITC-sinistrin) was administrated at a dose of 15 mg/100 g of body weight. Then, anesthesia was stopped, and the signal decay of the fluorescent agent was recorded for 1 h. The data obtained was analyzed with the specific software (MPD Studio Version RC15, MediBeacon) using a three-compartment model to obtain FITC-sinistrin half-life, which was converted to µl/100 g/min using a previously validated formula (Vergara et al., 2022; Giralt-López et al., 2020; Schreiber et al., 2012). For each mouse, the differences in GFR (ΔGFR) were calculated by subtracting the initial GFR from the final GFR.

### 2.5 Urine collection and UACR determination

Before (9–10 weeks of age) and at the end of the study (19–20 weeks of age), the first void spontaneous spot urine was obtained using an in-house urine multicollector for rodents (Patent U202131356 (ZBM reference U691ES00), Spanish Patents and Trades Office). Urinary albumin was determined by ELISA assay (Albuwell M, Ethos Biosciences, Logan Township, NJ, US), and creatinine was determined using a colorimetric assay based on the alkaline picrate method (The Creatinine Companion, Ethos Biosciences, Logan Township, NJ, United States). UACR was calculated from urine albumin and creatinine levels and expressed as µg albumin/mg creatinine.

### 2.6 Urine glucose measurement

Urine glucose was measured in spot urine samples obtained at the end of the experiment using a colorimetric enzymatic assay (Mouse Glucose Assay kit (81692, Crystal Chem)) as indicated in the manufacturer’s protocol. For the assay, db/db urine samples were previously diluted at 1:100 in Milli-Q water, whereas db/m urine samples were tested undiluted. Results were adjusted to blood glucose levels to normalize the values.

### 2.7 AST, ALT, and AP assessment

Serum levels of alanine transaminase (AST), alanine aminotransferase (ALT), and alkaline phosphatase (AP) were determined using an Atellica Solutions (Siemens) clinical chemistry analyzer. Hemolysed samples were rejected for the analysis.

### 2.8 Glomerular mesangial matrix expansion measurement

To assess mesangial matrix expansion, 10 representative cortical glomeruli from kidney sections were stained using periodic acid Schiff (PAS). Briefly, tissue sections of 4 µm were obtained from tissues embedded in paraffin blocks, deparaffinized in xylene, and rehydrated through graded alcohols. Then, the kidney slides were stained following the PAS staining protocol and using Schiff reagent (3952016, Millipore-Sigma). Samples were counterstained with Hematoxylin Gill N°3 solution (GHS332, Millipore-Sigma). The mesangial matrix areas from immunobiological images obtained at ×400 were analyzed by two independent, blinded observers using the ImageJ software (v1.53a, National Institutes of Health).

### 2.9 Podocyte density assessment

To determine the podocyte density in renal tissue, the expression of WT1 (a protein highly expressed in mature podocytes) was studied by immunohistochemistry (IHC). Blocks of paraffin-embedded kidney tissue were previously cut in 4 µm sections, deparaffinized using xylene, and rehydrated through graded alcohols. Endogenous peroxidases were blocked after deparaffination in an aqueous solution of 3% H_2_O_2_ and 10% methanol. Samples were boiled in citrate buffer (10 mM citric acid, pH 6.0) to retrieve the antigens and then blocked in 5% bovine serum albumin (BSA). The sections were incubated for 16 h at 4°C with an anti-WT1 primary antibody (Dilution 1:100, ab89901, Abcam). Biotinylated antibodies against rabbit IgGs (Dilution 1:250, BA-9500, Vector Laboratories) were used as secondary antibodies. Proteins were visualized by the colorimetric method through the avidin-biotin complex (ABC) peroxidase standard staining kit (32020, ThermoFisher Scientific) using the 3,3′-Diaminobenzidine (DAB) Enhanced Liquid Substrate System (D3939, Millipore-Sigma). Counterstaining of the slides was done with Hematoxylin Gill N°3 solution. Podocyte density (expressed as the number of podocytes per mm^2^ of glomerular area) was evaluated in 20 representative cortical glomeruli.

### 2.10 Renin detection by immunohistochemistry in kidneys

Renin protein expression was assessed by IHC following the abovementioned protocol. Anti-renin primary antibody was used at a dilution of 1:2000 (ab212197, Abcam). Once stained, five representative images of the juxtaglomerular apparatus per section were taken at ×400, and the renin-stained area was measured in µm^2^ using ImageJ software.

### 2.11 Collagen deposition assessment in the heart by PSR

Collagen deposition in the myocardium was evaluated in picrosirius red staining (PSR)-stained sections. Slides were previously deparaffinized, rehydrated, and subsequently counterstained for 15 min with 2.5 mg/mL Fast Green (11443054, Fisher Bioreagents) diluted in 1% acetic acid. Afterward, samples were washed and stained for 1 h with 0.1% Sirius Red (365548, Millipore-Sigma) diluted in saturated picric acid solution (P6744, Millipore-Sigma). Representative microphotographs were obtained at ×400 magnification (Olympus BX61 microscope). To quantify fibrosis, the collagen deposition was detected with an automated color recognition process plugin from ImageJ analysis software (V1.53a, National Institutes of Health). Perivascular and endocardial collagen was excluded.

### 2.12 Gene expression

Gene expression was analyzed by real-time qPCR using specific primers. Total RNA was previously isolated from tissues through phenol-guanidine thiocyanate-chloroform methodology using the commercial kit TRItidy G (A4051, AppliChem). A total of 1 µg of purified RNA was retrotranscribed to cDNA using the High-Capacity RNA-to-cDNA kit (4387406, ThermoFisher Scientific). mRNA expression was analyzed by RT-qPCR using PowerUp SYBR Green Master Mix (A25743, ThermoFisher Scientific). The ΔΔCt method was used to determine the relative expression of the gene using Hprt1 as a housekeeping gene. Vehicle-treated non-diabetic mice (db/m) were used as a reference group. All primer sequences are detailed in Supplementary Table S1.

### 2.13 ACE and ACE2 activities

To assess angiotensin-converting enzyme (ACE) and ACE2 activity measurement, kidney protein extracts were obtained from kidney cortex homogenized with lysis buffer (50 mM HEPES pH7.4, 150 mM NaCl, 0.5% Triton-X-100, 0.025 mM ZnCl_2_, 0.1 mM Pefabloc SC Plus (11873601001, Roche) and a 1:7 dilution of EDTA-free protease inhibitor cocktail (11836170001, Roche)). Samples were centrifuged at 16,000×G, 60 min, and 4°C, and the supernatant was recovered. The protein concentration was determined by a BCA protein assay kit (23225, ThermoFisher Scientific).

ACE activity was determined using N-hippuryl-L-histidyl-L-leucine (H1635, Millipore-Sigma) as was described previously (Roca-Ho et al., 2017). Renal cortical extracts previously quantified were adjusted to 0.5 μg/μL, and then 2 µL of each diluted extract was incubated with 73 µL test solution (0.4 M boric acid, 0.9 M NaCl pH 8.3) with the substrate N-hippuryl-L-histidyl-L-leucine for 25 min at 37°C. The reaction was stopped by adding 180 µL of 0.28 M NaOH. A 15 µL aliquot of o-phthalaldehyde (20 mg/mL in methanol) was added to each sample, which was then incubated for 10 min at room temperature and protected from light. The second reaction was stopped by the addition of 30 µL of 3 N-hydrochloric acid. Samples were then centrifuged at 800×G for 5 min, and 200 µL of the supernatant was retrieved and transferred into a 96-well black plate. Fluorescence was measured (Excitation 360 nm, Emission 485 nm) using Varioskan LUX Multimode Microplate Reader (ThermoFisher Scientific). All samples were measured in duplicate. A duplicate of 75 ng/μL of human recombinant ACE (SAE0075, Millipore-Sigma) and lysis buffer alone were used as a positive or negative control, respectively.

ACE2 activity was assessed through fluorescent enzymatic assay using ACE2-quenched fluorogenic substrate (Mca-Ala-Pro_lys (Dnp)-OH, BML-P163-0001, Enzo Life Sciences) adapting a method previously described (Roca-Ho et al., 2017). Briefly, 0.25 µg of kidney extract or 2.5 µL of serum were added to each well of a black 96-well plate. The reaction was performed using 5 µM (for kidney extracts) or 20 µM (for serum) of the fluorogenic substrate diluted in a solution containing several protease inhibitors (100 mM captropril, 5 µM bestatin, 5 µM amastatin, and 10 µM Z-Pro-prolinal). The plate was incubated for 4 h at 37°C, and fluorescence was detected (Excitation 320 nm, Emission 400 nm, 37°C) using the Varioskan LUX Multimode Microplate Reader (ThermoFisher Scientific). As positive and negative controls, 0.008 ng/μL of recombinant mouse ACE2 (3437-ZN-010, R&D Systems) and lysis buffer alone were used, respectively. The samples were also incubated with an ACE2 inhibitor (10 μM MLN-4760, Calbiochem). The relative fluorescence units (RFUs) were obtained using the ACE2 inhibitor subtracted from the RFUs obtained without the ACE2 inhibitor for each sample.

### 2.14 Statistical analysis

Data analysis was performed with Stata (V15.1, StataCorp LLC). Data are shown in tables as median and interquartile range (IQR) or box plots. First, a factorial ANOVA including sex, diabetes, empagliflozin, semaglutide, and empagliflozin–semaglutide interaction was performed. In the cases where residuals of the ANOVA model did not follow a normal distribution, the accuracy of the model was improved using a bootstrapping method. After ANOVA, *post hoc* multiple comparisons were performed using the Dunnet test when data followed a normal distribution and a Mann–Whitney test with Sidak corrected *p*-values in the other cases. Four multiple comparisons were performed between the vehicle-treated db/db groups and the remaining groups. *p*-values < 0.05 were considered significant.

## 3 Results

### 3.1 Empagliflozin, semaglutide, and their combination are safe and do not modify body weight or fat

After 8 weeks of treatment, we assessed the safety of the drugs by measuring hepatic enzymes AST, ALT, and AP in serum. As shown in Table 1, ALT and AP levels were higher in vehicle-treated db/db mice than in their non-diabetic littermates. The study drug combinations did not significantly increase serum AST, ALT, or AP in the db/db mice, indicating that the dosages used are safe. In addition, the db/db mice treated with RAM + EMP + SEM showed reduced serum AP compared to the vehicle-treated db/db mice (101 U/L, IQR: 90–114, vs. 160 U/L, IQR: 115.5–192.5, *p* < 0.001), suggesting that this drug combination improves the hepatic profile of the animals. After treatment, vehicle-treated db/db mice weighed 12.7 g (CI95%: 6.6–18.8) more than their non-diabetic littermates (Table 2). Treatment with empagliflozin, semaglutide, or their combination with ramipril did not change body weight in diabetic mice (Supplementary Figure S1A). In line with significantly higher body weight, vehicle-treated diabetic mice showed a significantly increased total body fat compared to non-diabetic controls (20.9 cm^3^ (IQR: 17.4–22.7) vs. 4.6 cm^3^ (IQR: 3.8–5.1), *p* < 0.001) (Table 1). The finding represents a 32.6% (CI95%: 28.4–36.9) increase in body fat driven by both higher subcutaneous and visceral fat. No differences were observed between vehicle db/db mice and the db/db mice treated with empagliflozin, semaglutide, or their combination. Sex had no effect in terms of body weight or fat.

**TABLE 1:** Serum alanine transaminase (AST), alanine aminotransferase (ALT), and alkaline phosphatase activity at the end of the experiment.

	db/m	db/db	db/db EMP + RAM	db/db SEM + RAM	db/db EMP + SEM + RAM
AST (U/L)	289 (IQR:210–627)	317.5 (IQR:181.5–575.5)	509 (IQR:366–616.5)	269 (IQR:189.3–392)	320 (IQR:210–619)
ALT (U/L)	57 (IQR:31–85)	103.5 (IQR:57.7–146.3)	144 (IQR:127–187)	126.5 (IQR:79.3–178)	86 (IQR:55–156)
Alkaline phosphatase (U/L)	75 (IQR:66–87)	160 (IQR:115.5–192.5)^$^	170 (IQR:159–183)	142 (IQR:111–161)	101 (IQR:90–114)^*^

**db/m**: non-diabetic mice treated with vehicle. Db/db: diabetic mice treated with vehicle. Db/db EMP + RAM: diabetic mice treated with empagliflozin and ramipril. Db/db SEM + RAM: diabetic mice treated with semaglutide and ramipril. Db/db EMP + SEM + RAM: diabetic mice treated with empagliflozin, semaglutide, and ramipril. Values are shown as median and interquartile range (Q1–Q3).

$*p* < 0.05 vehicle-treated db/db mice compared to vehicle-treated non-diabetic mice. *p < 0.05 db/db mice treated with empagliflozin, semaglutide, or their combination compared to vehicle-treated db/db mice.

**TABLE 2:** Body weight, body fat volume, and fat distribution at the end of the experiment.

	Db/m	db/db	db/db EMP + RAM	db/db SEM + RAM	db/db EMP + SEM + RAM
Body weight (g)	28.1 (IQR: 23.6–29.3)	40.8^$^ (IQR: 31.0–48.6)	43.3 (IQR: 39.3–45.3)	54.7 (IQR: 36.2–55.4)	47.3 (IQR: 39.5–52.7)
Total body fat (cm^3^)	4.6 (IQR: 3.8–5.1)	20.9^$^ (IQR: 17.4–22.7)	19.5 (IQR: 17.1–21.5)	24.3 (IQR: 23.5–24.6)	22.7 (IQR: 20.0–24.1)
Subcutaneous fat (cm^3^)	2.2 (IQR: 1.8–2.8)	11.9^$^ (IQR: 10.4–12.9)	11.9 (IQR: 10.2–12.7)	12.0 (IQR: 10.4–13.6)	12.1 (IQR: 10.3–13.8)
Abdominal fat (cm^3^)	2.1 (IQR: 1.7–2.5)	8.4^$^ (IQR: 6.9–9.5)	7.7 (IQR: 6.9–8.8)	10.9 (IQR: 10.3–11.5)	10.0 (IQR: 7.7–10.7)

db/m: non-diabetic mice treated with vehicle. Db/db: diabetic mice treated with vehicle. Db/db EMP + RAM: diabetic mice treated with empagliflozin and ramipril. Db/db SEM + RAM: diabetic mice treated with semaglutide and ramipril. Db/db EMP + SEM + RAM: diabetic mice treated with empagliflozin, semaglutide, and ramipril. Values are shown as median and interquartile range (Q1–Q3). ^**$**^ *p* < 0.05 vehicle-treated db/db mice compared to vehicle-treated non-diabetic mice. * *p* < 0.05 db/db mice treated with empagliflozin, semaglutide, or their combination compared to vehicle-treated db/db mice.

### 3.2 Empagliflozin combined with semaglutide exerts a greater glucose-lowering effect

**FIGURE 1:**
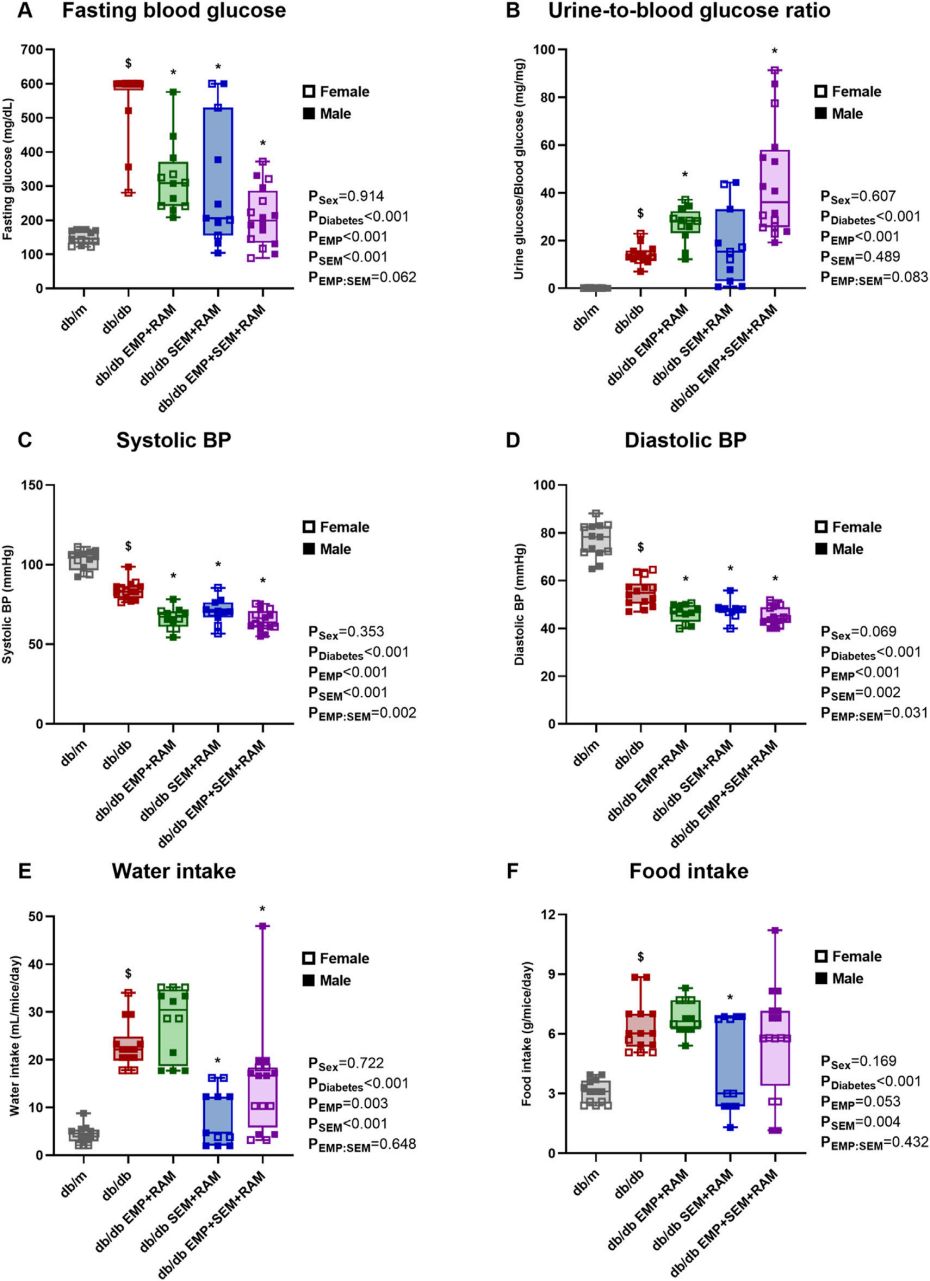
Metabolic parameters, blood pressure, and food intake after treatment in vehicle-treated db/m mice, vehicle-treated db/db mice, and db/db mice treated with empagliflozin, semaglutide, or their combination with ramipril. **(A)** 6-h fasting blood glucose measured in mg/dL. **(B)** Urine-to-blood glucose ratios were obtained simultaneously. **(C)** Systolic and **(D)** diastolic blood pressures measured after 8 weeks of treatment. **(E)** Water intake measured in mL/mouse per day. **(F)** Food intake measured in g/mouse per day. db/m: non-diabetic mice treated with vehicle. db/db: diabetic mice treated with vehicle. db/db EMP + RAM: diabetic mice treated with empagliflozin and ramipril. db/db SEM + RAM: diabetic mice treated with semaglutide and ramipril. db/db EMP + SEM + RAM: diabetic mice treated with empagliflozin, semaglutide, and ramipril. ^**$**^ *p* < 0.05 vehicle-treated db/db mice compared to vehicle treated non-diabetic mice. * *p* < 0.05 db/db mice treated with empagliflozin, semaglutide, or their combination compared to vehicle-treated db/db mice.

At the end of the experiment, vehicle-treated db/db mice showed significantly higher fasting blood glucose than non-diabetic controls (Figure 1A). Both empagliflozin and semaglutide reduced blood glucose in db/db mice after 8 weeks of treatment, but the combination of both decreased glycemia more quickly (Supplementary Figure S1B). In this sense, as expected, the combination of empagliflozin and semaglutide significantly decreased glucose by 112 mg/dL (CI95%: 36–190, *p* = 0.006) more than empagliflozin alone (Figure 1A). When measuring the urine glucose ratio, we observed that non-diabetic controls had a negligible loss of glucose (9 mg/dL, IQR: 8–13) compared to the overt glucosuria observed in db/db mice (7842 mg/dL, IQR: 7086–8701). Mice receiving the SGLT2i showed significantly higher glucose concentrations in the urine than diabetic controls, which is consistent with the characteristic glucosuric effect of this drug. Curiously, this effect was even higher in the EMP + SEM + RAM treatment group (Figure 1B). We did not find sex differences regarding blood glucose levels and glucosuria.

### 3.3 Ramipril decreased blood pressure in combination with empagliflozin and semaglutide

Vehicle-treated db/db mice showed lower systolic (18.9 mmHg lower, CI95%: 14.2–23.7, *p* < 0.001) and diastolic (21.6 mmHg lower, CI95%: 16.4–26.8, *p* < 0.001) blood pressures than non-diabetic controls (Figures 1C, D). Ramipril was administered for 8 weeks in the three groups of db/db mice receiving empagliflozin, semaglutide, or their combination. All three groups treated with ramipril showed lower blood pressure values than vehicle-treated diabetic mice, with a mean reduction between groups of 16.4 mmHg and 8.7 mmHg in systolic and diastolic BP, respectively (Figures 1C, D). BP reduction was similar between mice treated with empagliflozin, semaglutide, or their combination. There were no sex-related differences in BP.

### 3.4 Semaglutide reduced water intake but had less impact on food intake

Mice with diabetes showed an increased water and food intake with a statistically significant difference for diabetes as a factor (*p* < 0.001). At the end of the experiment, water was 18.6 mL/mouse/day (CI95%: 15.6–21.5) higher, and food intake was 3.2 g/mouse/day (CI95%: 2.4–4.0) higher (Figures 1E, F) in the vehicle-treated db/db mice than the non-diabetic controls. Mice treated with semaglutide and ramipril or empagliflozin and ramipril displayed a significant reduction in water intake compared to diabetic controls, with a mean reduction between groups of 11.5 mL/mouse/day (Figure 1E). However, when analyzing food intake, although there was a trend toward a decreased food consumption in both semaglutide groups, only the combination of semaglutide and ramipril significantly decreased food intake (1.9 g/mouse/day lower, CI95%: 0.25–3.6, *p* = 0.036) (Figure 1F). Sex had no impact on water or food intake.

### 3.5 The combination of empagliflozin, semaglutide, and ramipril reduced albuminuria and prevented diabetic glomerular hyperfiltration in male mice

**FIGURE 2:**
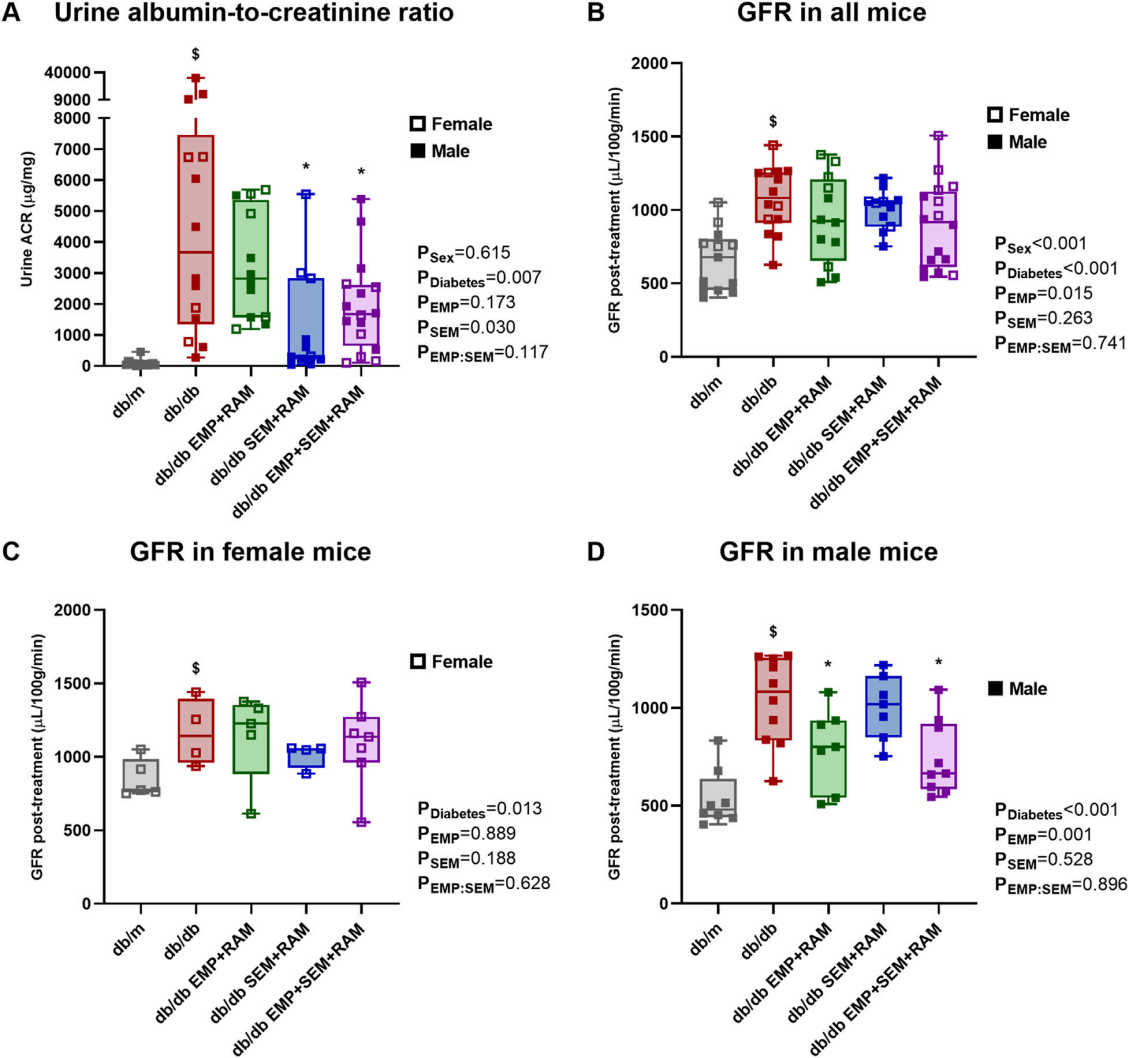
Kidney function parameters: urine albumin-to-creatinine ratio and glomerular filtration rate (GFR). **(A)** Urine albumin-to-creatinine ratio measured in μg/g. **(B)** GFR measured in μL per 100 g of body weight per minute at the end of the treatment in all mice included in the experiment. After 8 weeks of treatment, GFRs were different in **(C)** female and **(D)** male mice. db/m: non-diabetic mice treated with vehicle. db/db: diabetic mice treated with vehicle. db/db EMP + RAM: diabetic mice treated with empagliflozin and ramipril. db/db SEM + RAM: diabetic mice treated with semaglutide and ramipril. db/db EMP + SEM + RAM: diabetic mice treated with empagliflozin, semaglutide, and ramipril. ^**$**^ *p* < 0.05 vehicle-treated db/db mice compared to vehicle-treated non-diabetic mice. * *p* < 0.05 db/db mice treated with empagliflozin, semaglutide, or their combination compared to vehicle-treated db/db mice.

As expected, before starting the experiment, the db/db animals showed increased UACR compared to the db/m mice (Supplementary Figure S1C). At the end of the experiment, vehicle-treated mice with diabetes showed increased UACR (6579 μg/mg higher than db/m mice, CI95%: 1525–11632, *p* = 0.001) and GFR (418 μL/100 g/min higher than db/m mice, CI95%: 245–590, *p* < 0.001) compared to the non-diabetic controls (Figures 2A, B). The latter finding correlates with incipient diabetic nephropathy and glomerular hyperfiltration in db/db mice. When comparing the treated db/db mice with the diabetic control group, only the combinations of semaglutide with ramipril and empagliflozin, ramipril and semaglutide were able to reduce UACR to 5392 μg/mg (CI95%: 1066–9719, *p* = 0.010) and 4730 μg/mg (CI95%: 800–8660, *p* = 0.013), respectively (Figure 2A). Sex had no impact on UACR outcome.

There were no differences in GFR between control db/db mice and mice with diabetes receiving active treatments, but we observed a significant impact of sex as a factor in the GFR results (*p* < 0.001). In fact, GFR was 225 μL/100 g/min higher (CI95%: 96–355, *p* = 0.001) in female mice than in male mice when analyzing all the mice included in the experiment, and the finding was similar for both diabetic and non-diabetic mice. Following the analysis protocol, we evaluated the final GFR by sex (Figures 2C, D). In female mice, all groups with diabetes developed hyperfiltration, but treatment with empagliflozin, semaglutide, or their combination with ramipril did not affect GFR after 8 weeks of treatment (Figure 2C). However, in male mice, empagliflozin prevented glomerular hyperfiltration (*p* = 0.001 for empagliflozin as factor) with a significant decrease in GFR compared to diabetic controls of 243 μL/100 g/min (CI95%: 13–472, *p* = 0.037) for empagliflozin and ramipril combination, and 294 μL/100 g/min (CI95%: 92–496, *p* = 0.006) for the empagliflozin, semaglutide, and ramipril combination (Figure 2D).

### 3.6 The combination of empagliflozin, semaglutide, and ramipril reduces renal fibrosis and inflammation

**FIGURE 3:**
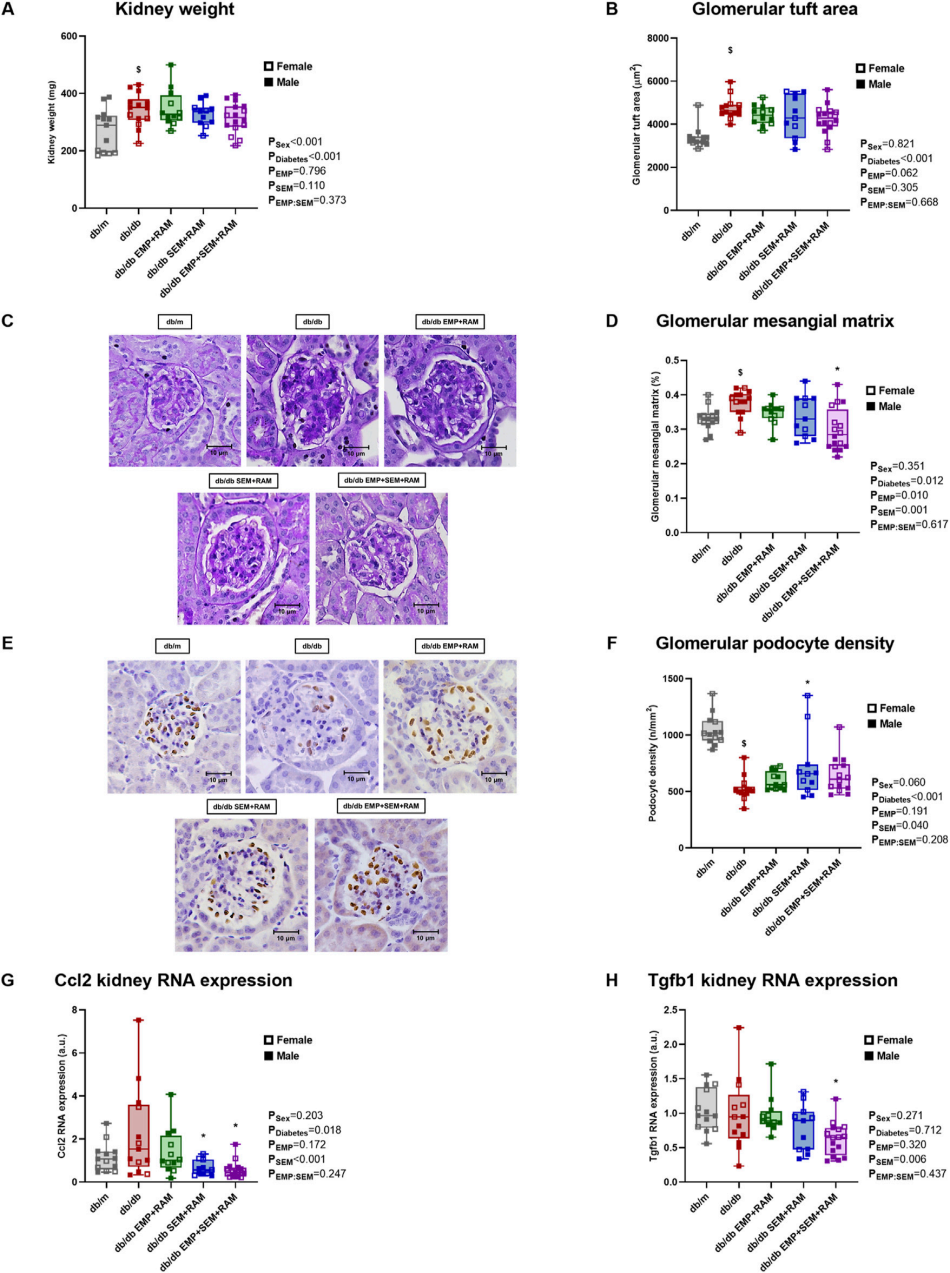
Kidney morphological studies and inflammatory and fibrotic kidney gene expression in vehicle-treated db/m mice, vehicle-treated db/db mice, and db/db mice treated with empagliflozin, semaglutide, or their combination with ramipril. **(A)** Kidney weight measured in mg. **(B)** Glomerular area and **(C)** representative microphotographs of PAS-stained glomeruli at ×400 magnification. **(D)** Glomerular mesangial matrix was also measured in PAS-stained kidney sections. **(E)** Representative microphotographs of WT-1 immunochemistry in glomeruli (×400 magnification) and **(F)** podocyte density measured through WT-1 staining. Kidney expression of **(G)** Ccl2 proinflammatory gene and **(H)** Tgfb1 profibrotic gene. db/m: non-diabetic mice treated with vehicle. db/db: diabetic mice treated with vehicle. db/db EMP + RAM: diabetic mice treated with empagliflozin and ramipril. db/db SEM + RAM: diabetic mice treated with semaglutide and ramipril. db/db EMP + SEM + RAM: diabetic mice treated with empagliflozin, semaglutide, and ramipril. ^**$**^ *p* < 0.05 vehicle-treated db/db mice compared to vehicle-treated non-diabetic mice. * *p* < 0.05 db/db mice treated with empagliflozin, semaglutide, or their combination compared to vehicle-treated db/db mice.

Diabetic mice treated with vehicle for 8 weeks showed increased kidney weight (72 mg higher, CI95%: 19–125, *p* = 0.039) and glomerular size (1371 μm^2^ greater area, CI95%: 973–1769, *p* < 0.001) compared to non-diabetic controls (Figures 3A, B), consistent with glomerular hyperfiltration. Kidneys were bigger in males than in females (kidney weight 73 mg higher, CI95%: 46–101), but the glomerular size was similar. Treatment with empagliflozin, semaglutide, or their combination with ramipril did not reduce kidney weight or glomerular size (Figures 3A, B). Additionally, vehicle-treated db/db mice showed a significant increase in glomerular mesangial matrix compared to non-diabetic mice (Figures 3C, D). Only the triple drug therapy with empagliflozin, semaglutide, and ramipril significantly reduced the increase in glomerular mesangial matrix compared to vehicle-treated db/db mice. Podocyte density was also significantly decreased in non-treated diabetic mice, with the latter being 518 podocytes per mm^2^ lower (CI95%: 422–614, *p* < 0.001) than in non-diabetic controls (Figures 3E, F). Only the combination of semaglutide and ramipril was able to preserve podocyte density (189 podocytes per mm^2^ higher, CI95%: 22–355, *p* = 0.021).

We also evaluated changes in the expression of genes related to proinflammatory and profibrotic pathways. Monocyte chemoattractant protein 1 (referred to in the figures as Ccl2 gene) is linked to proinflammatory states, and its renal expression was increased in diabetic mice (*p* = 0.018 for diabetes as a factor) (Figure 3G). Both mice groups treated with semaglutide showed significantly reduced Ccl2 gene expression (*p* = 0.004 and *p* = 0.001 for the SEM + RAM and EMP + SEM + RAM groups, respectively). Transforming growth factor β1 (referred to in the figures as the Tgfb1 gene) was not increased in a diabetic setting, but the combination therapy with empagliflozin, semaglutide, and ramipril significantly reduced this profibrotic gene RNA expression (*p* = 0.023) (Figure 3H), which correlates with the decreased mesangial matrix deposition in the same group of mice. Sex did not have a significant effect on any of these outcomes beyond kidney size.

### 3.7 The combination of empagliflozin, semaglutide, and ramipril prevents cardiomyocyte hypertrophy and heart fibrosis

**FIGURE 4:**
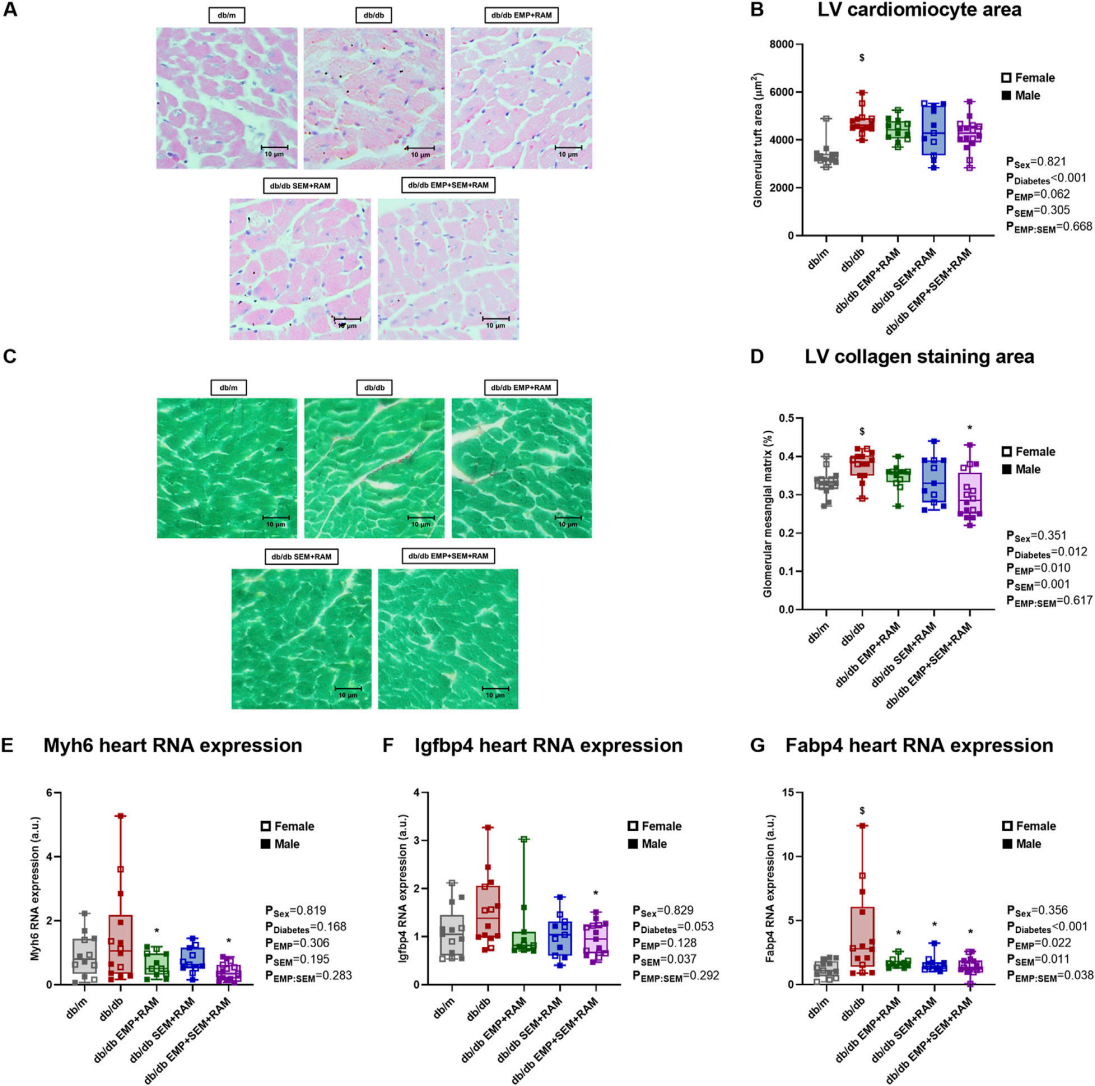
Heart morphological studies in vehicle-treated db/mmice, vehicle-treated db/db mice, and db/db mice treated with empagliflozin, semaglutide, or their combination with ramipril. **(A)** Representative microphotographs of left ventricle (LV) cardiomyocytes (×400 magnification) for measuring and **(B)** cardiomyocyte size as area (μm2). **(C)** Representative picrosirius red staining images on a fast green background (×400) for measuring **(D)** collagen deposition. db/m: non-diabetic mice treated with vehicle. db/db: diabetic mice treated with vehicle. Heart expression of **(E)** Myh7, **(F)** Igfbp4, and **(G)** Fabp4 genes. db/db EMP + RAM: diabetic mice treated with empagliflozin and ramipril. db/db SEM + RAM: diabetic mice treated with semaglutide and ramipril. db/db EMP + SEM + RAM: diabetic mice treated with empagliflozin, semaglutide, and ramipril. ^**$**^ *p* < 0.05 vehicle-treated db/db mice compared to vehicle-treated non-diabetic mice. * *p* < 0.05 db/db mice treated with empagliflozin, semaglutide, or their combination compared to vehicle-treated db/db mice.

After treatment, hearts were collected for histological examination. Diabetic mice treated with the vehicle showed an increased cardiomyocyte area in sections obtained from the left ventricle (LV). The cardiomyocyte area was higher in diabetic control mice than in non-diabetic mice (30 μm^2^, CI95%: 7–55, *p* = 0.001) (Figures 4A, B). Only the combination of empagliflozin, semaglutide, and ramipril was able to prevent LV hypertrophy compared to vehicle-treated db/db mice (LV cardiomyocyte area 27 μm^2^ lower, 95%CI: 9–45, *p* = 0.017). No significant differences were found between the db/m and the db/db groups when evaluating collagen deposition in LV by picrosirius red staining, although there was a trend toward an increased collagen deposition in vehicle-treated diabetic mice (Figures 4C, D). Even so, the triple therapy with empagliflozin, semaglutide, and ramipril decreased the collagen staining area in the LV by 38 μm^2^ (CI95%: 3–73, *p* = 0.030) compared to diabetic control mice (Figures 4C, D). In addition, we evaluated the gene expression of cardiac damage markers. The β-myosin heavy-chain (referred to in the figures as the Myh7 gene) is linked to cardiac function, and its expression tends to increase in diabetic mice (Figure 4E). All the drug combinations reduced Myh7 gene expression compared to the vehicle-treated db/db mice; however, the reduction was only significant in the combinations that included EMP (*p* = 0.039 and *p* = 0.003 for the EMP + RAM and EMP + SEM + RAM groups, respectively). The expressions of the cardiac damage markers insulin-like growth factor-binding protein 4 (IGFBP4) and fatty acid-binding protein 4 (FABP4) were increased in the diabetic setting with a significant difference for FABP4 when compared to non-diabetic mice (*p* < 0.001) (Figures 4F, G). While all the drug combinations significantly reduced the expression of FABP4 (Figure 4G), only the RAM + EMP + SEM combination was able to reduce cardiac overexpression of IGFBP4 in diabetic mice compared to vehicle-treated db/db mice (*p* = 0.027) (Figure 4F). Sex did not have a significant effect on any of the cardiac parameters analyzed.

### 3.8 Empagliflozin, semaglutide, or their combination with ramipril promotes the protective ACE2/Ang (1–7)/mas pathway in the RAS pathway

**FIGURE 5:**
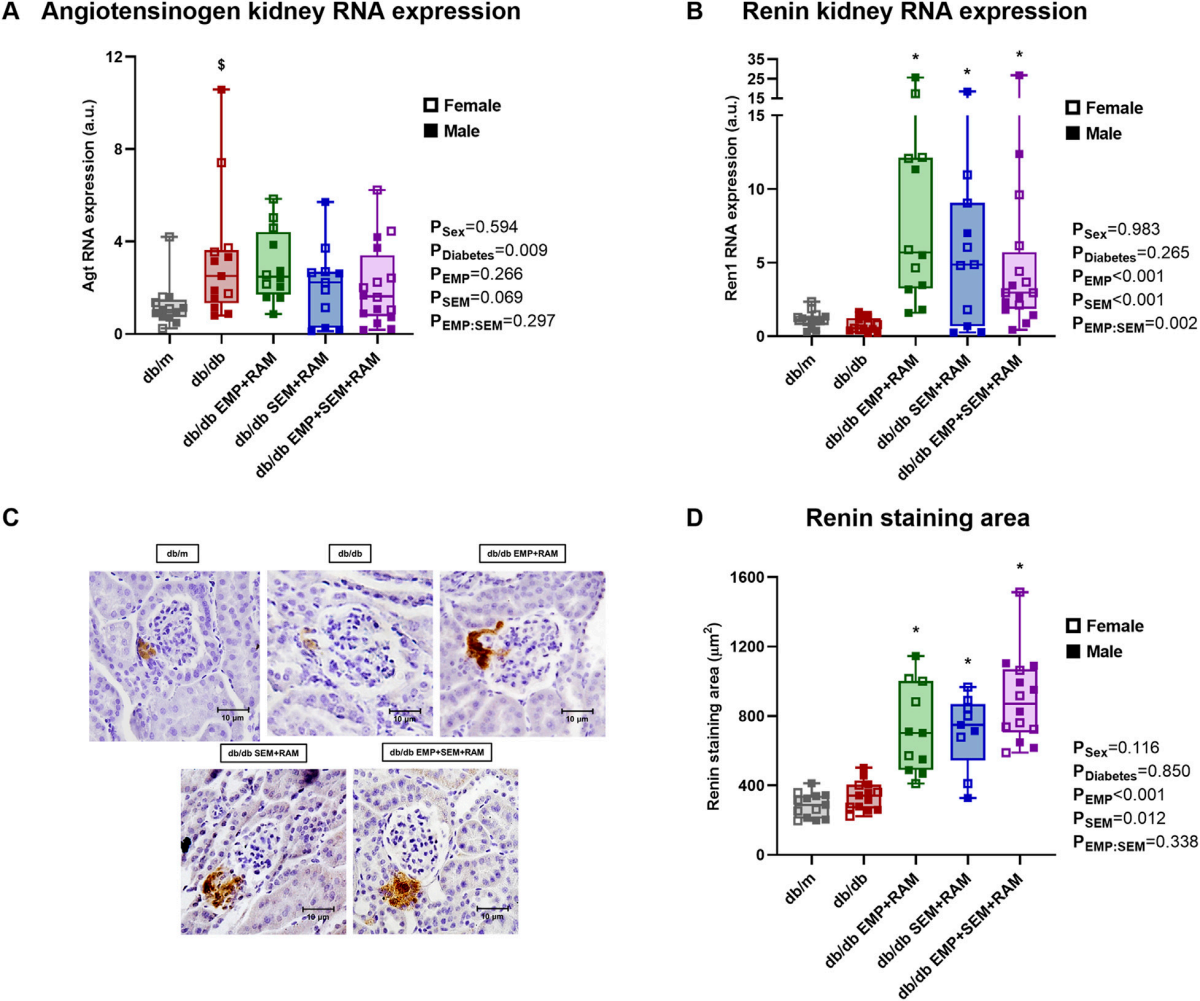
Angiotensinogen and renin expression in kidney cortex of vehicle-treated db/m mice, vehicle-treated db/db mice, and db/db mice treated with empagliflozin, semaglutide, or their combination with ramipril. Kidney cortex mRNA expression of **(A)** angiotensinogen and **(B)** renin. **(C)** Renin immunochemistry representative glomerular images obtained at ×400 magnification and **(D)** renin staining area (μm^2^) measured in the same images. db/m: non-diabetic mice treated with vehicle. db/db: diabetic mice treated with vehicle. db/db EMP + RAM: diabetic mice treated with empagliflozin and ramipril. db/db SEM + RAM: diabetic mice treated with semaglutide and ramipril. db/db EMP + SEM + RAM: diabetic mice treated with empagliflozin, semaglutide, and ramipril. ^**$**^ *p* < 0.05 vehicle-treated db/db mice compared to vehicle-treated non-diabetic mice. * *p* < 0.05 db/db mice treated with empagliflozin, semaglutide, or their combination compared to vehicle-treated db/db mice.

Aiming to identify the possible cardiorenal protective pathways of the combination of empagliflozin and semaglutide with ramipril, we measured the intrarenal RAS gene expression and activity in the kidney cortex. The first step in intrarenal RAS activation is angiotensinogen; its gene expression was significantly increased in vehicle-treated diabetic mice (2.5-fold higher, IQR: 1.5–3.5, *p* = 0.026) compared to non-diabetic controls. Angiotensinogen gene expression was found to increase in the three diabetic mice groups receiving treatments, and none of the drug combinations modified its expression (Figure 5A). Angiotensinogen is then converted to angiotensin I (Ang I) by renin, whose expression was not increased in non-treated diabetic mice when compared to the db/m control mice (Figure 5B). All of the three active treatments significantly increased renal renin expression with a mean increase between groups of 4.5-fold compared to db/m controls (*p* < 0.001, *p* = 0.046, and *p* < 0.001 for the EMP + RAM, SEM + RAM, and EMP + SEM + RAM groups, respectively) (Figure 5B) which may be ascribed to the ramipril component. In this line, the renin staining area was increased in the ramipril-treated db/db mice (Figures 5C, D), indicating a direct correlation between the renin gene and protein expression in the kidney.

**FIGURE 6:**
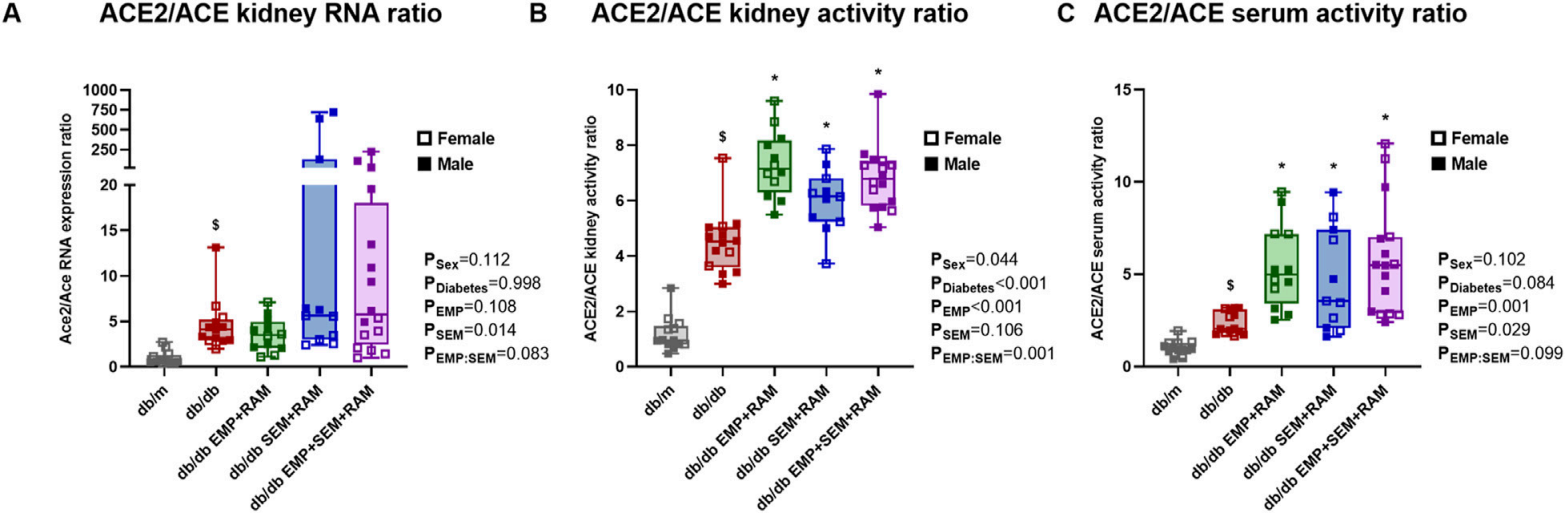
ACE2/ACE ratios in the kidney cortex and serum of vehicle-treated db/m mice, vehicle-treated db/db mice, and db/db mice treated with empagliflozin, semaglutide, or their combination with ramipril. **(A)** ACE2 and ACE mRNA expression and **(B)** ACE2/ACE activity ratios in the kidney cortex. **(C)** ACE2/ACE activity ratio in serum. Individual values of ACE and ACE2 gene expression and activity are shown in Supplementary Figure S2. db/m: non-diabetic mice treated with vehicle. db/db: diabetic mice treated with vehicle. db/db EMP + RAM: diabetic mice treated with empagliflozin and ramipril. db/db SEM + RAM: diabetic mice treated with semaglutide and ramipril. db/db EMP + SEM + RAM: diabetic mice treated with empagliflozin, semaglutide, and ramipril. ^**$**^ *p* < 0.05 vehicle-treated db/db mice compared to vehicle-treated non-diabetic mice. * *p* < 0.05 db/db mice treated with empagliflozin, semaglutide, or their combination compared to vehicle-treated db/db mice.

Finally, Ang I is converted to either angiotensin II (Ang II), which has profibrotic and proinflammatory effects, or Ang (1-7), which displays the opposite antifibrotic effects. The kidney conversion of Ang I toward the deleterious ACE/Ang II/angiotensin II type 1 receptor (AT_1_R) arm or the protective ACE2/angiotensin (1-7)/Mas arm depends on the activity of ACE or ACE2, respectively. In our experiment, when evaluating the ACE2/ACE RNA expression ratio after 8 weeks of treatment, ACE2 expression was increased over ACE (4.1 ACE2/ACE ratio, IQR: 2.9–4.9) in vehicle-treated db/db mice compared to non-diabetic controls (Figure 6A). In line with the gene expression, the ACE2/ACE renal activity ratio was significantly increased in vehicle-treated db/db mice (4.5 ACE2/ACE ratio, IQR: 3.6–5.0) compared to vehicle-treated db/m mice (0.9 ACE2/ACE ratio, IQR: 0.8–1.4) with a <0.001 *p*-value (Figure 6B). Additionally, mice groups receiving empagliflozin, semaglutide, or their combination with ramipril showed a significant increase of ACE2 renal activity over ACE with a mean ratio between groups of 6.7% and 32% increase in the ratio compared to the diabetic control group (Figure 6B). In concordance, the ACE2/ACE activity ratios in serum were also increased in db/db mice compared to non-diabetic db/m mice (Figure 6C). Similarly, the db/db mice treated with empagliflozin and/or semaglutide in combination with a RAS blocker showed increased serum ACE2/ACE ratio (*p* = 0.002, *p* = 0.026, and *p* < 0.001 for EMP + RAM, SEM + RAM and EMP + SEM + RAM groups, respectively) (Figure 6C). We did not find gender differences in the intrarenal RAS profile analysis.

## 4 Discussion

In this study, we explored the protective effect of empagliflozin (an SGLT2i) and/or semaglutide (a GLP-1RA) with a RAS blocker on kidney and heart of uninephrectomized db/db mice. Despite the lower BP and decreased ACE levels observed in the db/db mouse model (Vergara et al., 2022), we included ramipril in all treatment combinations to better mimic human DKD, as a RAS blocker is the first line of treatment. In addition, most RCTs designed to test the renoprotective effect of SGLT2i or GLP-1RA in patients with DKD maintained the RAS blocker as the standard of care (Wanner et al., 2016; Perkovic et al., 2019; Neal et al., 2017; Bernard et al., 2015). Further, we included both female and male mice to explore the potential sex-specific effects of the active treatment combinations studied.

We first addressed the safety of the drug combinations by measuring hepatic enzymes AST, ALT, and AP in serum. ALT and AP levels were increased in vehicle-treated db/db mice, which may be related to steatohepatitis (Qiao et al., 2022). The study drug combinations did not significantly increase the hepatic enzymes in the db/db mice, demonstrating that the therapies administrated are safe. Additionally, the combination of RAM + EMP + SEM reduced serum AP, suggesting that this drug combination improves the hepatic profile of the animals. In this sense, both SGLT2i and GLP-1RA have been shown to improve steatohepatitis in diabetic mouse models (Qiao et al., 2022; Niu et al., 2022). All drug combinations tested contained a blood glucose-lowering agent; thus, as expected, they all improved glycemic control. However, the hypoglycemic effect was maximized when empagliflozin and semaglutide were administered in combination with ramipril, mirroring results obtained in RCTs (Fulcher et al., 2016; Frías et al., 2016). This may be attributed to the fact that EMP + SEM + RAM-treated db/db mice tended to have higher glucose levels in urine than EMP + RAM-treated animals, suggesting that semaglutide further promoted the glucosuric effect of empagliflozin. A possible explanation for this enhanced glucosuric effect is the ability of the GLP-1RA to inhibit sodium-hydrogen exchanger 3 (NHE3) (Farah et al., 2016; Crajoinas et al., 2011) as its function is also dependent on SGLT2 activity (Pessoa et al., 2014). In any case, this finding is interesting as semaglutide, when administered alone with ramipril, does not induce glucosuria in db/db mice, which is consistent with its different mechanism of action (García-Carro et al., 2019).

The db/db mice treated with semaglutide alone or in combination with empagliflozin and a RAS blocker showed decreased water and food intake compared to vehicle-treated db/db mice, which demonstrates the efficacy of the GLP-1RA in our experimental setting. However, the decrease in food intake produced by semaglutide was variable between animals; this effect could be explained because the db/db mice model is deficient in the leptin receptor and therefore avoids satiety (Giralt-López et al., 2020). This reason may also explain why no treatment was able to decrease body weight or body fat distribution. Further, all active treatments produced a similar decrease in BP in db/db mice, which was expected due to the ramipril component.

Regarding renal protection, semaglutide (alone or combined with empagliflozin and a RAS blocker) significantly reduced UACR compared to vehicle-treated db/db mice. This effect was not observed in the EMP + RAM group, suggesting that semaglutide had a higher impact in reducing UACR than empagliflozin in our experimental setting. Curiously, none of the treatments affected the GFR in female db/db mice, but male db/db mice treated with EMP + RAM or EMP + SEM + RAM showed improvement in hyperfiltration. This finding is consistent with decreased mesangial matrix expansion and recovery of the podocyte density in both groups of db/db mice that were treated with semaglutide. Interestingly, mesangial matrix expansion was only significantly reduced in the EMP + SEM + RAM group, while podocyte density recovery was only significant in the SEM + RAM group. These discrepancies may be related to the inclusion of both male and female mice in our study.

Although beneficial effects of the SGLT2i and the GLP-1RA have been observed in both men and women with DKD (Heerspink et al., 2019; Wanner et al., 2016; Sorli et al., 2017; Neal et al., 2017; Wiviott et al., 2019), data from several studies have shown some differential effects of the SGLT2i and the GLP-1RA related to sex in terms of reduction of major adverse cardiovascular events (MACE) or the individual components of the MACE composite (Sharma et al., 2023; Singh and Singh, 2020; Diallo et al., 2022). In a 5-year follow-up study, SGLT2i reduced MACE in men, in men and women over 65 years, in men with a history of heart failure, and in women with baseline atherosclerotic cardiovascular disease. In the same study, GLP-1RAs seemed to reduce the rate of all-cause mortality only in men over 65 years of age (Sharma et al., 2023). Results from a metanalysis revealed that GLP-1RA reduced MACE in both men and women, while SGLT-2i only produced a significant MACE reduction in men (Singh and Singh, 2020). Contrarily, in another metaanalysis, no gender differences were noted in terms of MACE reduction in DKD patients treated with either SGLT2i or GLP-1RA (Diallo et al., 2022).

Whether the protective effect of the SGLT2i and GLP-1RA is different in men and women with diabetes is unclear as the results vary between studies. This may be related to the presence of selection bias, confounders, or inadequate statistical power (underrepresentation of women), among others. However, we have observed gender differences in a mouse model of type 2 diabetes that suggest that the renal protection exerted by the study drugs may be higher in men.

We also analyzed heart cardiomyocyte area and fibrosis, which are increased in the diabetic milieu. Only the triple therapy with EMP + SEM + RAM significantly decreased cardiomyocyte hypertrophy and heart fibrosis, suggesting that this combination is superior to EMP + RAM and SEM + RAM in cardiac protection. In addition, the combination of EMP + SEM + RAM decreased the expression of several cardiac damage markers (Myh7, IGFBP4, and FABP4). Although empagliflozin (Arow et al., 2020; Li et al., 2019; Quagliariello et al., 2021) and semaglutide (Withaar et al., 2023; Husain et al., 2019) have been shown to protect the heart in experimental diabetic models, in a previous study of our group, we only observed improvement in cardiac parameters when administering empagliflozin combined with ramipril and atrasentan to db/db mice (Vergara et al., 2022).

Finally, we have studied the intrarenal RAS as it plays an important role in DKD (Clotet-Freixas et al., 2018). In agreement with previous studies (Shin et al., 2016), kidney AGT was increased in mice with diabetes consistent with intrarenal activation of the RAS; however, this effect was not reversed with any treatment. Although we did not find increased renin expression in the vehicle-treated mice with diabetes, which contrasts with our previous report (Vergara et al., 2022), all tested drug combinations increased renin gene and protein expression, probably related to the ramipril component. This effect is well known and may be ascribed to ACE inhibition resulting in the interruption of the negative feedback mechanisms and to the lower BP, causing reduced renal blood flow and stimulation of glomerular afferent arteriole baroreceptors (Gomez et al., 1988; Gomez et al., 1990). The activation of the classical RAS (via ACE activity) promotes inflammation and fibrosis, but the non-classical pathway (via ACE2 activity) protects against diabetic nephropathy (Mori et al., 2014; Oudit et al., 2010; Soler et al., 2007). Despite decreased kidney ACE activity in db/db mice, which is thought to represent an early compensatory effect that opposes the diabetes-mediated increase in tubular Ang II(71,72), ramipril decreased its activity both in serum and in the kidney, consistent with its biological action. In this sense, ACE2 activity was not significantly modified by the effect of the therapies, which resulted in a higher renal ACE2/ACE activity ratio in the treated animals. This may imply that the increased kidney RAS activation in diabetic mice noted by increased AGT, and fueled by ramipril-induced renin production, was redirected toward the non-classical RAS pathway (ACE2/Ang/Mas) (1-7) by ramipril with no differences between treatments. These findings suggest that all the therapeutic combinations tested promote the non-classical RAS pathway, whose add-on tissue protection eventually affects proinflammatory or profibrotic pathways or oxidative stress involved in DKD progression (Oudit et al., 2010; Zhang et al., 2016).

However, as in every study, this work is not exempt from limitations. First, we only tested EMP and SEM with RAM and did not include the monotherapies to ensure enough statistical power during *post hoc* comparisons. This design may limit the interpretation of the results, as we do not have data about the effect of the compounds alone in our experimental setting. Second, the uninephrectomized db/db mouse model may not totally represent human DKD. The db/db mice show decreased BP compared to their non-diabetic littermates, as depicted in our results. This must be considered when translating our results to patients with diabetes and CKD who are mostly hypertensive. In addition, the db/db mice are leptin deficient, which may limit the interpretation of the results obtained with semaglutide. Further, the dosages used are not the same as those used in clinical practice, which also challenges the translation of the results. Third, water intake was increased in the animals treated with RAM + EMP compared to the animals in groups RAM + SEM and RAM + EMP + SEM. As a result, the db/db RAM + EMP mice may be receiving more ramipril. However, all the combinations decrease ACE activity and BP similarly, suggesting an equal biological effect of ramipril in all the experimental groups with no evident ramipril overdosing effects in the RAM + EMP group. Finally, we analyzed some markers to assess heart tissue damage, but no other cardiac functional studies, such as echocardiography, were performed.

## 5 Conclusions

In conclusion, in the present study, we characterized the impact of combining empagliflozin and semaglutide with a RAS blocker in a clinically relevant mouse model of type 2 diabetes. We demonstrated that empagliflozin combined with semaglutide with a RAS blocker is superior in lowering blood glucose, decreasing glomerular hyperfiltration in male mice, and reducing glomerular mesangial matrix expansion compared with either drug alone with a RAS blocker. Further, only the combination of empagliflozin and semaglutide with a RAS blocker decreased cardiomyocyte hypertrophy and heart fibrosis in db/db mice. Our results suggest a synergic effect of the SGLT2i and the GLP-1RA in cardiorenal protection in db/db mice with DKD.

## Data Availability

The raw data supporting the conclusions of this article will be made available by the authors, without undue reservation.

## References

[B1] Adamson C. Docherty K. F. Heerspink H. J. L. de Boer R. A. Damman K. Inzucchi S. E. (2022). Initial decline (dip) in estimated glomerular filtration rate after initiation of dapagliflozin in patients with heart failure and reduced ejection fraction: insights from DAPA-HF. Circulation 146 (6), 438–449. 10.1161/CIRCULATIONAHA.121.058910 35442064 PMC9354593

[B2] Anker S. D. Butler J. Filippatos G. Ferreira J. P. Bocchi E. Böhm M. (2021). Empagliflozin in heart failure with a preserved ejection fraction. N. Engl. J. Med. 385 (16), 1451–1461. 10.1056/NEJMoa2107038 34449189

[B3] Arow M. Waldman M. Yadin D. Nudelman V. Shainberg A. Abraham N. G. (2020). Sodium-glucose cotransporter 2 inhibitor Dapagliflozin attenuates diabetic cardiomyopathy. Cardiovasc Diabetol. 19 (1), 7. 10.1186/s12933-019-0980-4 31924211 PMC6953156

[B4] Bakris G. L. Agarwal R. Anker S. D. Pitt B. Ruilope L. M. Rossing P. (2020). Effect of finerenone on chronic kidney disease outcomes in type 2 diabetes. N. Engl. J. Med. 383 (23), 2219–2229. 10.1056/NEJMoa2025845 33264825

[B5] Bernard Z. Christoph W. Lachin J. M. David F. Erich B. Stefan H. (2015). Empagliflozin, cardiovascular outcomes, and mortality in type 2 diabetes. N. Engl. J. Med. 373 (22), 2117–2128. 10.1056/NEJMoa1504720 26378978

[B6] Clotet-Freixas S. Soler M. J. Palau V. Anguiano L. Gimeno J. Konvalinka A. (2018). Sex dimorphism in ANGII-mediated crosstalk between ACE2 and ACE in diabetic nephropathy. Lab. Invest 98 (9), 1237–1249. 10.1038/s41374-018-0084-x 29884907

[B7] Crajoinas R. O. Oricchio F. T. Pessoa T. D. Pacheco B. P. M. Lessa L. M. A. Malnic G. (2011). Mechanisms mediating the diuretic and natriuretic actions of the incretin hormone glucagon-like peptide-1. Am. J. physiology Ren. physiology 301 (2), F355–F363. 10.1152/ajprenal.00729.2010 21593184

[B8] De Nicola L. Gabbai F. B. Liberti M. E. Sagliocca A. Conte G. Minutolo R. (2014). Sodium/glucose cotransporter 2 inhibitors and prevention of diabetic nephropathy: targeting the renal tubule in diabetes. Am. J. Kidney Dis. 64 (1), 16–24. 10.1053/j.ajkd.2014.02.010 24673844

[B9] de Zeeuw D. Coll B. Andress D. Brennan J. J. Tang H. Houser M. (2014). The endothelin antagonist atrasentan lowers residual albuminuria in patients with type 2 diabetic nephropathy. J. Am. Soc. Nephrol. 25 (5), 1083–1093. 10.1681/ASN.2013080830 24722445 PMC4005314

[B10] Diallo A. Carlos-Bolumbu M. Galtier F. (2022). Age, sex, race, BMI, and duration of diabetes differences in cardiovascular outcomes with glucose lowering drugs in type 2 diabetes: a systematic review and meta-analysis. EClinicalMedicine 54, 101697. 10.1016/j.eclinm.2022.101697 36263397 PMC9574412

[B11] Farah L. X. S. Valentini V. Pessoa T. D. Malnic G. McDonough A. A. Girardi A. C. C. (2016). The physiological role of glucagon-like peptide-1 in the regulation of renal function. Am. J. Physiology-Renal Physiology 310 (2), F123–F127. 10.1152/ajprenal.00394.2015 PMC5504384 26447224

[B12] Fernandez-Fernandez B. Fernandez-Prado R. Górriz J. L. Martinez-Castelao A. Navarro-González J. F. Porrini E. (2019). Canagliflozin and renal events in diabetes with established nephropathy clinical evaluation and study of diabetic nephropathy with atrasentan: what was learned about the treatment of diabetic kidney disease with canagliflozin and atrasentan? Clin. Kidney J. 12 (3), 313–321. 10.1093/ckj/sfz070 31198532 PMC6543971

[B13] Frías J. P. Guja C. Hardy E. Ahmed A. Dong F. Öhman P. (2016). Exenatide once weekly plus dapagliflozin once daily versus exenatide or dapagliflozin alone in patients with type 2 diabetes inadequately controlled with metformin monotherapy (DURATION-8): a 28 week, multicentre, double-blind, phase 3, randomised controlled trial. Lancet Diabetes Endocrinol. 4 (12), 1004–1016. 10.1016/S2213-8587(16)30267-4 27651331

[B14] Fulcher G. Matthews D. R. Perkovic V. de Zeeuw D. Mahaffey K. W. Mathieu C. (2016). Efficacy and safety of canagliflozin when used in conjunction with incretin-mimetic therapy in patients with type 2 diabetes. Diabetes Obes. Metab. 18 (1), 82–91. 10.1111/dom.12589 26450639

[B15] García-Carro C. Vergara A. Agraz I. Jacobs-Cachá C. Espinel E. Seron D. (2019). The new era for reno-cardiovascular treatment in type 2 diabetes. J. Clin. Med. 8 (6), 864. 10.3390/jcm8060864 31212945 PMC6617211

[B16] Gembardt F. Bartaun C. Jarzebska N. Mayoux E. Todorov V. T. Hohenstein B. (2014). The SGLT2 inhibitor empagliflozin ameliorates early features of diabetic nephropathy in BTBR ob/ob type 2 diabetic mice with and without hypertension. Am. J. Physiol. Ren. Physiol. 307 (3), F317–F325. 10.1152/ajprenal.00145.2014 24944269

[B17] Gerstein H. C. Colhoun H. M. Dagenais G. R. Diaz R. Lakshmanan M. Pais P. (2019). Dulaglutide and renal outcomes in type 2 diabetes: an exploratory analysis of the REWIND randomised, placebo-controlled trial. Lancet 394 (10193), 131–138. 10.1016/S0140-6736(19)31150-X 31189509

[B18] Giralt-López A. Molina-Van den Bosch M. Vergara A. García-Carro C. Seron D. Jacobs-Cachá C. (2020). Revisiting experimental models of diabetic nephropathy. Int. J. Mol. Sci. 21 (10), 3587. 10.3390/ijms21103587 32438732 PMC7278948

[B19] Gomez R. A. Chevalier R. L. Everett A. D. Elwood J. P. Peach M. J. Lynch K. R. (1990). Recruitment of renin gene-expressing cells in adult rat kidneys. Am. J. Physiol. 259 (4 Pt 2), F660–F665. 10.1152/ajprenal.1990.259.4.F660 2221104

[B20] Gomez R. A. Lynch K. R. Chevalier R. L. Everett A. D. Johns D. W. Wilfong N. (1988). Renin and angiotensinogen gene expression and intrarenal renin distribution during ACE inhibition. Am. J. Physiol. 254 (6 Pt 2), F900–F906. 10.1152/ajprenal.1988.254.6.F900 2837909

[B21] Górriz J. L. Navarro-González J. F. Ortiz A. Vergara A. Nuñez J. Jacobs-Cachá C. (2020a). Sodium-glucose cotransporter 2 inhibition: towards an indication to treat diabetic kidney disease. Nephrol. Dial. Transpl. 35 (Suppl. 1), i13–i23. 10.1093/ndt/gfz237 PMC6993197 32003834

[B22] Górriz J. L. Soler M. J. Navarro-González J. F. García-Carro C. Puchades M. J. D’Marco L. (2020b). GLP-1 receptor agonists and diabetic kidney disease: a call of attention to nephrologists. J. Clin. Med. 9 (4), 947. 10.3390/jcm9040947 32235471 PMC7231090

[B23] Griffin M. Rao V. S. Ivey-Miranda J. Fleming J. Mahoney D. Maulion C. (2020). Empagliflozin in heart failure: diuretic and cardiorenal effects. Circulation 142 (11), 1028–1039. 10.1161/CIRCULATIONAHA.120.045691 32410463 PMC7521417

[B24] Heerspink H. J. L. Kiyosue A. Wheeler D. C. Lin M. Wijkmark E. Carlson G. (2023). Zibotentan in combination with dapagliflozin compared with dapagliflozin in patients with chronic kidney disease (ZENITH-CKD): a multicentre, randomised, active-controlled, phase 2b, clinical trial. Lancet 402 (10416), 2004–2017. 10.1016/S0140-6736(23)02230-4 37931629

[B25] Heerspink H. J. L. Kohan D. E. de Zeeuw D. (2021). New insights from SONAR indicate adding sodium glucose co-transporter 2 inhibitors to an endothelin receptor antagonist mitigates fluid retention and enhances albuminuria reduction. Kidney Int. 99 (2), 346–349. 10.1016/j.kint.2020.09.026 33144213

[B26] Heerspink H. J. L. Parving H. H. Andress D. L. Bakris G. Correa-Rotter R. Hou F. F. (2019). Atrasentan and renal events in patients with type 2 diabetes and chronic kidney disease (SONAR): a double-blind, randomised, placebo-controlled trial. Lancet 393 (10184), 1937–1947. 10.1016/S0140-6736(19)30772-X 30995972

[B27] Husain M. Birkenfeld A. L. Donsmark M. Dungan K. Eliaschewitz F. G. Franco D. R. (2019). Oral semaglutide and cardiovascular outcomes in patients with type 2 diabetes. N. Engl. J. Med. 381 (9), 841–851. 10.1056/NEJMoa1901118 31185157

[B28] Jiang P. Sun N. Yang W. Xiao L. Zhou L. Gu B. (2023). Development of a novel Fc fusion protein dual glucagon-like peptide-1 and gastric inhibitory polypeptide receptor agonists. Diabetes Obes. Metab. 25 (11), 3356–3365. 10.1111/dom.15235 37580307

[B29] Kidney Disease: Improving Global Outcomes KDIGO Diabetes Work Group (2022). KDIGO 2022 clinical practice guideline for diabetes management in chronic kidney disease. Kidney Int. 102 (5S), S1–S127. 10.1016/j.kint.2022.06.008 36272764

[B30] Kraus B. J. Weir M. R. Bakris G. L. Mattheus M. Cherney D. Z. I. Sattar N. (2021). Characterization and implications of the initial estimated glomerular filtration rate “dip” upon sodium-glucose cotransporter-2 inhibition with empagliflozin in the EMPA-REG OUTCOME trial. Kidney Int. 99 (3), 750–762. 10.1016/j.kint.2020.10.031 33181154

[B31] Li C. Zhang J. Xue M. Li X. Han F. Liu X. (2019). SGLT2 inhibition with empagliflozin attenuates myocardial oxidative stress and fibrosis in diabetic mice heart. Cardiovasc Diabetol. 18 (1), 15. 10.1186/s12933-019-0816-2 30710997 PMC6359811

[B32] Lorenzo M. Jacobs-Cachá C. Palau P. Amiguet M. Seller J. Núñez E. (2023). Short-term changes in peak VO2 after initiation of dapagliflozin in heart failure across iron status. JACC Heart Fail 11 (11), 1611–1622. 10.1016/j.jchf.2023.07.010 37676213

[B33] Maekawa M. Maekawa T. Sasase T. Takagi K. Takeuchi S. Kitamoto M. (2022). Pathophysiological analysis of uninephrectomized db/db mice as a model of severe diabetic kidney disease. Physiol. Res. 71 (2), 209–217. 10.33549/physiolres.934784 35344670 PMC9150550

[B34] Mann J. F. E. Ørsted D. D. Brown-Frandsen K. Marso S. P. Poulter N. R. Rasmussen S. (2017). Liraglutide and renal outcomes in type 2 diabetes. N. Engl. J. Med. 377 (9), 839–848. 10.1056/nejmoa1616011 28854085

[B35] Marso S. P. Daniels G. H. Brown-Frandsen K. Kristensen P. Mann J. F. E. Nauck M. A. (2016). Liraglutide and cardiovascular outcomes in type 2 diabetes. N. Engl. J. Med. 375 (4), 311–322. 10.1056/nejmoa1603827 27295427 PMC4985288

[B36] Martínez-Díaz I. Martos N. Llorens-Cebrià C. Álvarez F. J. Bedard P. W. Vergara A. (2023). Endothelin receptor antagonists in kidney disease. Int. J. Mol. Sci. 24 (4), 3427. 10.3390/ijms24043427 36834836 PMC9965540

[B37] McMurray J. J. V. Solomon S. D. Inzucchi S. E. Køber L. Kosiborod M. N. Martinez F. A. (2019). Dapagliflozin in patients with heart failure and reduced ejection fraction. N. Engl. J. Med. 381 (21), 1995–2008. 10.1056/NEJMoa1911303 31535829

[B38] Mori J. Patel V. B. Ramprasath T. Alrob O. A. DesAulniers J. Scholey J. W. (2014). Angiotensin 1-7 mediates renoprotection against diabetic nephropathy by reducing oxidative stress, inflammation, and lipotoxicity. Am. J. Physiol. Ren. Physiol. 306 (8), F812–F821. 10.1152/ajprenal.00655.2013 24553436

[B39] Mosenzon O. Wiviott S. D. Cahn A. Rozenberg A. Yanuv I. Goodrich E. L. (2019). Effects of dapagliflozin on development and progression of kidney disease in patients with type 2 diabetes: an analysis from the DECLARE-TIMI 58 randomised trial. Lancet Diabetes Endocrinol. 7 (8), 606–617. 10.1016/S2213-8587(19)30180-9 31196815

[B40] Navale A. M. Paranjape A. N. (2016). Glucose transporters: physiological and pathological roles. Biophys. Rev. 8 (1), 5–9. 10.1007/s12551-015-0186-2 PMC5425736 28510148

[B41] Neal B. Perkovic V. Mahaffey K. W. de Zeeuw D. Fulcher G. Erondu N. (2017). Canagliflozin and cardiovascular and renal events in type 2 diabetes. N. Engl. J. Med. 377 (7), 644–657. 10.1056/NEJMoa1611925 28605608

[B42] Nestor J. J. Parkes D. Feigh M. Suschak J. J. Harris M. S. (2022). Effects of ALT-801, a GLP-1 and glucagon receptor dual agonist, in a translational mouse model of non-alcoholic steatohepatitis. Sci. Rep. 12 (1), 6666. 10.1038/s41598-022-10577-2 35461369 PMC9035150

[B43] Ninichuk V. Kulkarni O. Clauss S. Anders H. J. (2007). Tubular atrophy, interstitial fibrosis, and inflammation in type 2 diabetic db/db mice. An accelerated model of advanced diabetic nephropathy. Eur. J. Med. Res. 12 (8), 351–355. 17933712

[B44] Niu S. Chen S. Chen X. Ren Q. Yue L. Pan X. (2022). Semaglutide ameliorates metabolism and hepatic outcomes in an NAFLD mouse model. Front. Endocrinol. (Lausanne) 13, 1046130. 10.3389/fendo.2022.1046130 36568109 PMC9780435

[B45] Oudit G. Y. Liu G. C. Zhong J. Basu R. Chow F. L. Zhou J. (2010). Human recombinant ACE2 reduces the progression of diabetic nephropathy. Diabetes 59 (2), 529–538. 10.2337/db09-1218 19934006 PMC2809962

[B46] Perkovic V. Jardine M. J. Neal B. Bompoint S. Heerspink H. J. L. Charytan D. M. (2019). Canagliflozin and renal outcomes in type 2 diabetes and nephropathy. N. Engl. J. Med. 380 (24), 2295–2306. 10.1056/NEJMoa1811744 30990260

[B47] Perkovic V. Tuttle K. R. Rossing P. Mahaffey K. W. Mann J. F. E. Bakris G. (2024). Effects of semaglutide on chronic kidney disease in patients with type 2 diabetes. N. Engl. J. Med. 391, 109–121. 10.1056/NEJMoa2403347 38785209

[B48] Pessoa T. D. Campos L. C. G. Carraro-Lacroix L. Girardi A. C. C. Malnic G. (2014). Functional role of glucose metabolism, osmotic stress, and sodium-glucose cotransporter isoform-mediated transport on Na+/H+ exchanger isoform 3 activity in the renal proximal tubule. J. Am. Soc. Nephrol. JASN. 25 (9), 2028–2039. 10.1681/ASN.2013060588 24652792 PMC4147971

[B49] Pitt B. Filippatos G. Agarwal R. Anker S. D. Bakris G. L. Rossing P. (2021). Cardiovascular events with finerenone in kidney disease and type 2 diabetes. N. Engl. J. Med. 385 (24), 2252–2263. 10.1056/NEJMoa2110956 34449181

[B50] Qiao P. Jia Y. Ma A. He J. Shao C. Li X. (2022). Dapagliflozin protects against nonalcoholic steatohepatitis in db/db mice. Front. Pharmacol. 13, 934136. 10.3389/fphar.2022.934136 36059948 PMC9437261

[B51] Quagliariello V. De Laurentiis M. Rea D. Barbieri A. Monti M. G. Carbone A. (2021). The SGLT-2 inhibitor empagliflozin improves myocardial strain, reduces cardiac fibrosis and pro-inflammatory cytokines in non-diabetic mice treated with doxorubicin. Cardiovasc Diabetol. 20 (1), 150. 10.1186/s12933-021-01346-y 34301253 PMC8305868

[B52] Roca-Ho H. Riera M. Palau V. Pascual J. Soler M. J. (2017). Characterization of ACE and ACE2 expression within different organs of the NOD mouse. Int. J. Mol. Sci. 18 (3), 563. 10.3390/ijms18030563 28273875 PMC5372579

[B53] Ruggenenti P. Perticucci E. Cravedi P. Gambara V. Costantini M. Sharma S. K. (2008). Role of remission clinics in the longitudinal treatment of CKD. J. Am. Soc. Nephrol. 19 (6), 1213–1224. 10.1681/ASN.2007090970 18354029 PMC2396935

[B54] Schreiber A. Shulhevich Y. Geraci S. Hesser J. Stsepankou D. Neudecker S. (2012). Transcutaneous measurement of renal function in conscious mice. Am. J. Physiol. Ren. Physiol. 303 (5), F783–F788. 10.1152/ajprenal.00279.2012 22696603

[B55] Sharma A. Wood S. Bell J. S. Blasio M. J. D. Ilomäki J. Ritchie R. H. (2023). Sex differences in risk of cardiovascular events and mortality with sodium glucose co-transporter-2 inhibitors versus glucagon-like peptide 1 receptor agonists in Australians with type 2 diabetes: a population-based cohort study. Lancet Regional Health - West. Pac. 33, 100692. 10.1016/j.lanwpc.2023.100692 PMC10166999 37181530

[B56] Shin S. J. Chung S. Kim S. J. Lee E. M. Yoo Y. H. Kim J. W. (2016). Effect of sodium-glucose Co-transporter 2 inhibitor, dapagliflozin, on renal renin-angiotensin system in an animal model of type 2 diabetes. PLoS One 11 (11), e0165703. 10.1371/journal.pone.0165703 27802313 PMC5089752

[B57] Singh A. K. Singh R. (2020). Gender difference in cardiovascular outcomes with SGLT-2 inhibitors and GLP-1 receptor agonist in type 2 diabetes: a systematic review and meta-analysis of cardio-vascular outcome trials. Diabetes Metab. Syndr. 14 (3), 181–187. 10.1016/j.dsx.2020.02.012 32142999

[B58] Škrtić M. Cherney D. Z. I. (2015). Sodium-glucose cotransporter-2 inhibition and the potential for renal protection in diabetic nephropathy. Curr. Opin. Nephrol. Hypertens. 24 (1), 96–103. 10.1097/MNH.0000000000000084 25470017

[B59] Soler M. J. Wysocki J. Ye M. Lloveras J. Kanwar Y. Batlle D. (2007). ACE2 inhibition worsens glomerular injury in association with increased ACE expression in streptozotocin-induced diabetic mice. Kidney Int. 72 (5), 614–623. 10.1038/sj.ki.5002373 17579661

[B60] Sorli C. Harashima S. I. Tsoukas G. M. Unger J. Karsbøl J. D. Hansen T. (2017). Efficacy and safety of once-weekly semaglutide monotherapy versus placebo in patients with type 2 diabetes (SUSTAIN 1): a double-blind, randomised, placebo-controlled, parallel-group, multinational, multicentre phase 3a trial. Lancet Diabetes Endocrinol. 5 (4), 251–260. 10.1016/S2213-8587(17)30013-X 28110911

[B61] Szablewski L. (2017). Distribution of glucose transporters in renal diseases. J. Biomed. Sci. 24 (1), 64. 10.1186/s12929-017-0371-7 28854935 PMC5577680

[B62] Vergara A. Jacobs-Cacha C. Llorens-Cebria C. Ortiz A. Martinez-Diaz I. Martos N. (2022). Enhanced cardiorenal protective effects of combining SGLT2 inhibition, endothelin receptor antagonism and RAS blockade in type 2 diabetic mice. Int. J. Mol. Sci. 23 (21), 12823. 10.3390/ijms232112823 36361612 PMC9656616

[B63] Vergara A. Jacobs-Cachá C. Soler M. J. (2019). Sodium-glucose cotransporter inhibitors: beyond glycaemic control. Clin. Kidney J. 12 (3), 322–325. 10.1093/ckj/sfz019 31198226 PMC6543973

[B64] Vergara A. Llorens-Cebrià C. Martos N. Martínez-Díaz I. Stein F. Domínguez-Báez P. (2023). The membrane-associated protein 17 (MAP17) is up-regulated in response to empagliflozin on top of RAS blockade in experimental diabetic nephropathy. Clin. Sci. (Lond). 137 (1), 87–104. 10.1042/CS20220447 36524468

[B65] Wanner C. Inzucchi S. E. Lachin J. M. Fitchett D. von Eynatten M. Mattheus M. (2016). Empagliflozin and progression of kidney disease in type 2 diabetes. N. Engl. J. Med. 375 (4), 323–334. 10.1056/NEJMoa1515920 27299675

[B66] Wenzel R. R. Littke T. Kuranoff S. Jürgens C. Bruck H. Ritz E. (2009). Avosentan reduces albumin excretion in diabetics with macroalbuminuria. J. Am. Soc. Nephrol. 20 (3), 655–664. 10.1681/ASN.2008050482 19144760 PMC2653691

[B67] Withaar C. Meems L. M. G. Nollet E. E. Schouten E. M. Schroeder M. A. Knudsen L. B. (2023). The cardioprotective effects of semaglutide exceed those of dietary weight loss in mice with HFpEF. JACC Basic Transl. Sci. 8 (10), 1298–1314. 10.1016/j.jacbts.2023.05.012 38094687 PMC10714176

[B68] Wiviott S. D. Raz I. Bonaca M. P. Ofri M. Kato E. T. Avivit C. (2019). Dapagliflozin and cardiovascular outcomes in type 2 diabetes. N. Engl. J. Med. 380 (4), 347–357. 10.1056/NEJMoa1812389 30415602

[B69] Wysocki J. Ye M. Soler M. J. Gurley S. B. Xiao H. D. Bernstein K. E. (2006). ACE and ACE2 activity in diabetic mice. Diabetes 55 (7), 2132–2139. 10.2337/db06-0033 16804085

[B70] Ye M. Wysocki J. Naaz P. Salabat M. R. LaPointe M. S. Batlle D. (2004). Increased ACE 2 and decreased ACE protein in renal tubules from diabetic mice: a renoprotective combination? Hypertension 43 (5), 1120–1125. 10.1161/01.HYP.0000126192.27644.76 15078862

[B71] Zander M. Madsbad S. Madsen J. L. Holst J. J. (2002). Effect of 6-week course of glucagon-like peptide 1 on glycaemic control, insulin sensitivity, and beta-cell function in type 2 diabetes: a parallel-group study. Lancet 359 (9309), 824–830. 10.1016/S0140-6736(02)07952-7 11897280

[B72] Zhang F. Ren X. Zhao M. Zhou B. Han Y. (2016). Angiotensin-(1-7) abrogates angiotensin II-induced proliferation, migration and inflammation in VSMCs through inactivation of ROS-mediated PI3K/Akt and MAPK/ERK signaling pathways. Sci. Rep. 6, 34621. 10.1038/srep34621 27687768 PMC5043354

[B73] Zinman B. Bhosekar V. Busch R. Holst I. Ludvik B. Thielke D. (2019). Semaglutide once weekly as add-on to SGLT-2 inhibitor therapy in type 2 diabetes (SUSTAIN 9): a randomised, placebo-controlled trial. Lancet Diabetes Endocrinol. 7 (5), 356–367. 10.1016/S2213-8587(19)30066-X 30833170

