# Supplementary material for: SGLT2i and GLP1-RA exert additive cardiorenal protection with a RAS blocker in uninephrectomized db/db mice
Source: Front Pharmacol. 2024 Oct 7;15:1415879. doi: 10.3389/fphar.2024.1415879 (PMC11491409; doi:10.3389/fphar.2024.1415879)
Supplement: Supplementary file 1 [file DataSheet1.docx]

Supplementary Material

SGLT2i combined with GLP1-RA exert additive cardiorenal protection on top of RAS blockade in uninephrectomized db/db mice

Nerea Martos-Guillami^1,^ †, Ander Vergara^1,2,^ †, Carmen Llorens-Cebrià^1^, Aku Enam Motto^1,3^, Irene Martínez-Díaz^1^, Francisco Gonçalves ^1^,Maria Magdalena Garcias-Ramis^4^, Estibaliz Allo- Urzainqui^4^, Alonso Narváez^5^, Sheila Bermejo^1,2^, Vicent Muñoz^1^, Juan León-Román^1^, Roser Ferrer-Costa^4^, Conxita Jacobs-Cachá^1,2,4,*^, Jordi Vilardell-Vilà^1,*^, María José Soler^1,2,*^

^1^ Nephrology and Transplantation Research Group. Vall d’Hebron Institut de Recerca (VHIR), Vall d’Hebron Hospital Universitari, Vall d’Hebron Barcelona Hospital Campus. Barcelona, Spain.

^2^ Redes de Investigación Cooperativa Orientadas a Resultados en Salud (RICORS). Instituto de Salud Carlos III (RD21/0005/0031).

^3^ Laboratory of Physiology/ Pharmacology, Unit of Pathophysiology, Bioactive Substances and Safety, Faculty of Sciences, University of Lomé, BP 1515, Togo.

^4^ Clinical Biochemistry Department. Vall d’Hebron Hospital Universitari, Vall d’Hebron Barcelona Hospital Campus. Barcelona, Spain.

^5^ Urology Department. Vall d’Hebron Hospital Universitari, Vall d’Hebron Barcelona Hospital Campus. Barcelona, Spain.

†These authors have contributed equally and share first authorship

# Supplementary Tables

Supplementary Table S1. **Primer sequences used for SYBR Green real-time quantitative PCR (RT-qPCR).** The present table displays protein and gene names for each of the genes analyzed the forward and reverse primer sequence, as well as the cDNA dilution employed in each case.

| **Protein** | **Gene** | **Forward primer (5’-3’)** | **Reverse primer (5’-3’)** | **cDNA dilution** |
| --- | --- | --- | --- | --- |
| Angiotensin converting enzyme | Ace | CGCCGCTATGGGGACAAATA | ATGTCTCCCAGCAAATGGGC | 1/25 |
| Angiotensin converting enzyme 2 | Ace2 | CGCAGAGATCAAGCCATTGT | TCCATCAACTTCCTCCTCACA | 1/25 |
| Angiotensinogen | Agt | CGTGCCCCTAGGTGAGAGAG | TCCAAGTCAGGAGGTCGTTC | 1/25 |
| β-myosin heavy-chain (β-MHC) | Myh7 | CTCAAGCTGCTCAGCAATCTATTT | GGAGCGCAAGTTTGTCATAAGT | 1/50 |
| Fatty acid-binding protein 4 (FABP4) | Fabp4 | ATGATCATCAGCGTAAATGG | GCCTTTCATAACACATTCCA | 1/25 |
| Hypoxanthine-guanine phosphoribosyltransferase | Hprt1 | TGTTGTTGGATATGCCCTTG | AATGACACAAACGTGATTCAAA | 1/25 |
| Insulin-like growth factor-binding protein 4 (IGFBP4) | Igfbp4 | TACCCACGAAGACCTCTTCATC | GTCTTCCGATCCACACACCA | 1/25 |
| Monocyte chemoattractant protein 1 | Ccl2 | AGGTCCCTGTCATGCTTCTG | CGTTAACTGCATCTGGCTGA | 1/25 |
| Renin | Ren1 | ACCTTGCTTGTGGGATTCAC | CCTGATCCGTAGTGGATGGT | 1/25 |
| Transforming growth factor β1 | Tgfb1 | TGAGTGGCTGTCTTTTGACG | AGCCCTGTATTCCGTCTCCT | 1/25 |

# Supplementary Figures

**
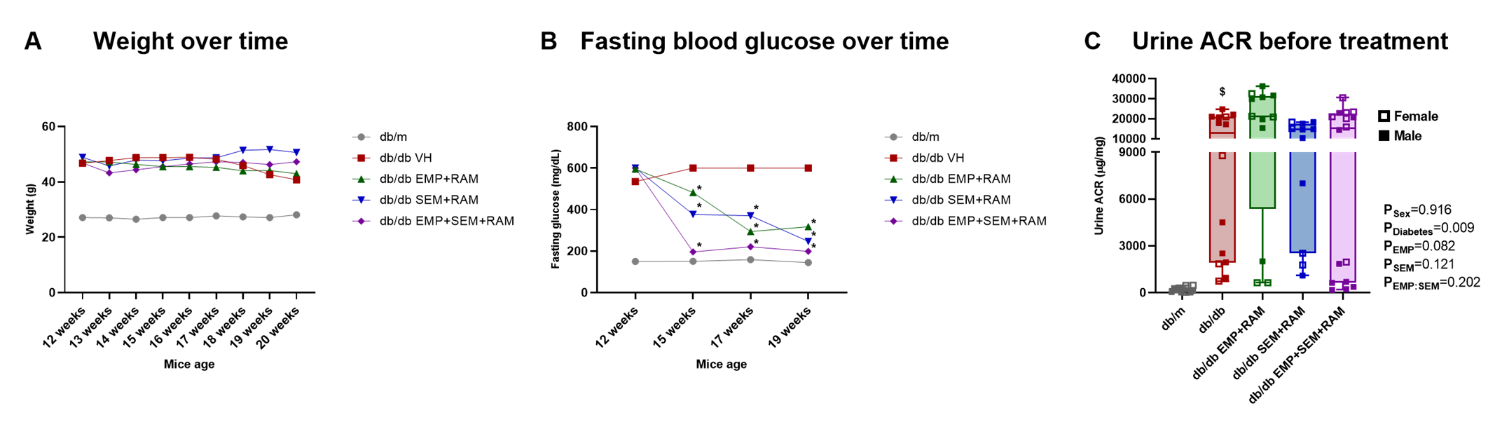
**

**Figure S1.** **(A) Weight over time, (B) fasting blood glucose over time and (C) UACR before treatment in vehicle-treated db/m mice, vehicle-treated db/db mice, and db/db mice treated with empagliflozin, semaglutide or their combination on top of ramipril. db/m**: non-diabetic mice treated with vehicle. **db/db**: diabetic mice treated with vehicle. **db/db EMP+RAM**: diabetic mice treated with empagliflozin and ramipril. **db/db SEM+RAM**: diabetic mice treated with semaglutide and ramipril. **db/db EMP+SEM+RAM**: diabetic mice treated with empagliflozin, semaglutide and ramipril. **^$^**p<0.05 vehicle-treated db/db mice compared to vehicle-treated non-diabetic mice. *****p<0.05 db/db mice treated with empagliflozin, semaglutide or their combination compared to vehicle-treated db/db mice.

**
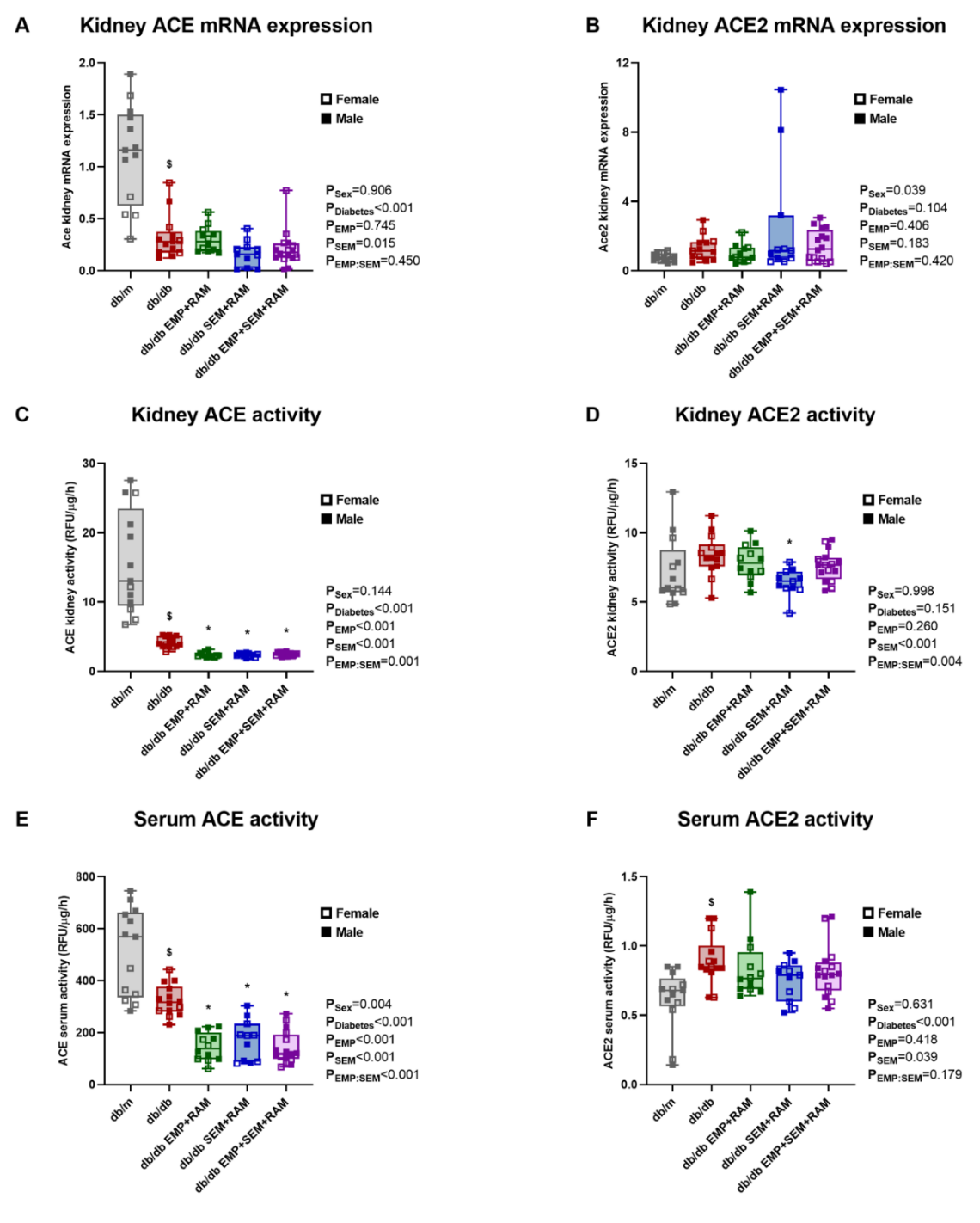
**

**Figure S2**. **ACE and ACE2 kidney gene expression and activities in kidney cortex and serum of vehicle-treated db/m mice, vehicle-treated db/db mice, and db/db mice treated with empagliflozin, semaglutide or their combination on top of ramipril. (A)** ACE and (**B**) ACE2 mRNA expression in the kidney cortex. (**C**) ACE and **(D)** ACE2 activity in kidney. (**E**) ACE and **(F)** ACE2 activity in serum. **db/m**: non-diabetic mice treated with vehicle. **db/db**: diabetic mice treated with vehicle. **db/db EMP+RAM**: diabetic mice treated with empagliflozin and ramipril. **db/db SEM+RAM**: diabetic mice treated with semaglutide and ramipril. **db/db EMP+SEM+RAM**: diabetic mice treated with empagliflozin, semaglutide and ramipril. **^$^**p<0.05 vehicle-treated db/db mice compared to vehicle-treated non-diabetic mice. *****p<0.05 db/db mice treated with empagliflozin, semaglutide or their combination compared to vehicle-treated db/db mice.
